# Genome-wide survey of two-component signal transduction systems in the plant growth-promoting bacterium *Azospirillum*

**DOI:** 10.1186/s12864-015-1962-x

**Published:** 2015-10-22

**Authors:** Stéphanie Borland, Anne Oudart, Claire Prigent-Combaret, Céline Brochier-Armanet, Florence Wisniewski-Dyé

**Affiliations:** Université de Lyon, Université Lyon 1, CNRS, UMR5557, Laboratoire d’Ecologie Microbienne, 43 7 boulevard du 11 novembre 1918, F-69622 Villeurbanne, France; Université de Lyon, Université Lyon 1, CNRS, UMR5558, Laboratoire de Biométrie et Biologie Evolutive, 43 boulevard du 11 novembre 1918, F-69622 Villeurbanne, France

**Keywords:** *Azospirillum*, Hybrid histidine kinase, PGPR, Signal transduction, Two-component systems

## Abstract

**Background:**

Two-component systems (TCS) play critical roles in sensing and responding to environmental cues. *Azospirillum* is a plant growth-promoting rhizobacterium living in the rhizosphere of many important crops. Despite numerous studies about its plant beneficial properties, little is known about how the bacterium senses and responds to its rhizospheric environment. The availability of complete genome sequenced from four *Azospirillum* strains (*A. brasilense* Sp245 and CBG 497, *A. lipoferum* 4B and *Azospirillum* sp. B510) offers the opportunity to conduct a comprehensive comparative analysis of the TCS gene family.

**Results:**

*Azospirillum* genomes harbour a very large number of genes encoding TCS, and are especially enriched in hybrid histidine kinases (HyHK) genes compared to other plant-associated bacteria of similar genome sizes. We gained further insight into HyHK structure and architecture, revealing an intriguing complexity of these systems. An unusual proportion of TCS genes were orphaned or in complex clusters, and a high proportion of predicted soluble HKs compared to other plant-associated bacteria are reported. Phylogenetic analyses of the transmitter and receiver domains of *A. lipoferum* 4B HyHK indicate that expansion of this family mainly arose through horizontal gene transfer but also through gene duplications all along the diversification of the *Azospirillum* genus. By performing a genome-wide comparison of TCS, we unraveled important ‘genus-defining’ and ‘plant-specifying’ TCS.

**Conclusions:**

This study shed light on *Azospirillum* TCS which may confer important regulatory flexibility. Collectively, these findings highlight that *Azospirillum* genomes have broad potential for adaptation to fluctuating environments.

**Electronic supplementary material:**

The online version of this article (doi:10.1186/s12864-015-1962-x) contains supplementary material, which is available to authorized users.

## Background

Plants live in intimate association with complex communities of microorganisms that fulfill important functions relative to plant growth and health [1]. These microorganisms can establish beneficial, neutral or detrimental associations of varying intimacy with their hosts. Plant-associated bacteria in the rhizosphere (*i.e.* the soil region strongly influenced by root activity), the phyllosphere (*i.e.* the aerial parts of plants dominated by the leaves) and endosphere (*i.e.* internal tissues) evolve in highly heterogeneous habitats, influenced by plant activity, variation of physicochemical factors and other microbial activities [2]. Therefore, the need to cope with various conditions and the ability to respond to environmental stimuli is essential for plant-associated bacteria.

Two component systems (TCS) are one of the primary means used by bacteria to sense and adjust their behaviour accordingly. They play important roles in a broad range of adaptive mechanisms such as virulence, chemotaxis, metabolism, motility, etc. [3]. The TCS signalling pathway relies on a phosphotransfer reaction between two proteins, a generally membrane-bound histidine kinase (HK), characterized by the presence of a HisKA (PF00512) and a HATPase domain (PF02518), and a response regulator (RR) containing a REC domain (PF00072). In the most basic scheme, upon detection of a signal on the *N*-terminal variable region containing the input domain, the HK autophosphorylates on a conserved histidine residue and subsequently transfers the phosphoryl group to a conserved aspartyl residue at the receiver domain (REC) of the RR. Phosphorylation of the RR leads to activation of the downstream output domain that elicits specific responses. More complex versions of TCS exist, called hybrid HKs (HyHK), with HK fused to REC, allowing multiple intramolecular phosphotransfer reactions or phosphorelays [4].

The growing number of complete prokaryotic genomes sequenced to date has dramatically improved our knowledge of TCS prevalence among bacteria. The number of TCS genes within an organism varies greatly and a strong relationship between bacterial ecological niche and sophistication of organism behaviour has been highlighted [5, 6]. In plant pathogens where the TCS gene repertoire has been well studied, their number varies from 46 in *Erwinia amylovora* Ea1189 to 124 in *Pseudomonas syringae* (3 strains of various pathovars) [7, 8]. Nonetheless, despite extensive knowledge on TCS genes, still very little is known about these systems in beneficial plant-associated bacteria.

The alphaproteobacterium *Azospirillum* is a plant growth-promoting bacteria (PGPR) living in the rhizosphere of numerous plants such as crops and grasses [9, 10]. Successful interaction with its host plant includes root colonization (motility, chemotaxis, and biofilm formation), phytohormone production, nitrogen fixation, modulation of plant hormonal balance, etc. Although several essential mechanisms underlying its positive association with plants have been elucidated, their utilization at the field-scale has been hampered by its inconstancy in terms of plant yields [11]. Indeed, *Azospirillum* are found associated with a large range of host plants, and have been isolated from many soil types under distinct geographic locations. Differential varietal response upon *Azospirillum* inoculation has been reported for several crops strongly suggesting the existence of host specificity [12, 13]. Recently, with the availability of genomic sequences of four different *Azospirillum* strains, comparative genomic analyses have been implemented [14, 15]. However the functions of a large proportion of their genes remain largely unknown [14, 15].

In this work, we focused on the analysis of *Azospirillum* TCS repertoire. We assessed their distribution among the four strains and unveiled the prevalence of this multigenic family among this genus, especially when considering HyHKs. We further gained insight into the structure and architecture of HyHKs, revealing an intriguing complexity of these systems. Phylogenetic analyses of *A. lipoferum* 4B HyHKs underlined the importance of horizontal gene transfer and gene duplication events in the expansion of these HyHKs in this strain. Finally, we highlighted TCS potentially involved in host plant specificity.

## Methods

### Data collection and sequence information

TCS protein sequences were retrieved from the MaGe genome annotation platform (https://www.genoscope.cns.fr/agc/microscope/home/index.php) for *Azospirillum* strains and from the P2CS database (http://p2cs.org/) for other strains [16, 17]. Detailed survey of *Azospirillum* TCS using P2CS was done on three complete genomes of *Azospirillum*: *A. lipoferum* 4B, *Azospirillum* sp. B510 and *A. brasilense* Sp245. For the fourth genome *A. brasilense* CBG497 (that can be accessed at the following link: https://www.genoscope.cns.fr/agc/microscope/home/index.php), the analysis was carried out manually following the P2CS pipeline to allow accurate comparisons between the four strains studied [17].

### Identification of TCS genes

All TCS retrieved were further classified as HK, RR or phosphotransfer protein (*i.e.* presence of HisKA or Hpt domain –PF01627- and absence of REC or HATPase domain). Additionally, HKs were classified as classic or hybrid according to the presence of a REC domain within the protein. TCS classified by P2CS as ‘Mispredicted’ were included in our study and those classified as ‘Incomplete HK’ were manually validated based on the following criteria: (i) the presence of both HisKA domain (containing the His residue or H-box) and HATPase; (ii) exceptions were made for HKs whose transmitter region was composed solely of a HisKA domain but if its gene was adjacent to that encoding another TCS gene. Finally, all the identified putative TCS were manually curated to avoid false-positive and false-negative results.

### Comparison of TCS content among plant-associated bacteria

To compare *Azospirillum* TCS genes with plant-associated bacteria, proteomes of plant-associated bacteria were selected among those available in P2CS database in September 2014. As very few intraspecies differences in TCS gene content were found, one representative of each species was selected in this study, *i.e.* 85 proteomes. They included 34 PGPR (*i.e.* free-living beneficial bacteria including non-obligatory endophytes), 25 symbionts (*i.e.* obligatory endophytes and root-nodulating bacteria) and 26 phytopathogens (*i.e.* causing plant diseases) (Additional file 1: Table S1). Eight genomes of non-plant-associated bacteria were also included, *i.e.* those of the model bacteria, *Bacillus subtilis* subsp*. subtilis* str. 168*, Escherichia coli* K-12*, Myxoccocus xanthus* DK 1622, a species whose TCS gene repertoire is well documented [18] and five genomes of bacteria belonging to the *Rhodospirillaceae* family (two *Magnetospirillum* and three *Rhodospirillum* strains), that are the closest *Azospirillum* relatives [14].

### Detection of orthologous TCS

In order to identify orthologous proteins, a best reciprocal BLASTP analysis was performed using each TCS protein of the four *Azospirillum* strains as seed and the four corresponding proteomes as target as previously described [15]. The following parameters were considered: amino acid identity percentage greater than 35 % and query coverage greater than 75 %. To confirm the results obtained with reciprocal BLAST, we used the protein sequence clustering program CD-HIT at 35 % sequence identity level [19].

### Phylogenetic analysis

A phylogenetic tree showing the relatedness of the different 97 organisms (85 plant-associated bacteria + 3 model species + 5 close relatives of *Azospirillum* + 4 *Azospirillum* strains) discussed below has been constructed using ten ribosomal proteins (L3, L5, L11, L13, L14, S3, S7, S9, S11, and S17). The protein sequences were aligned with ClustalΩ [20]. Resulting multiples alignments were visually inspected using SEAVIEW 4.5.4., then trimmed using the Gblocks program with default parameters and finally concatenated [21, 22]. A distance-based method phylogenetic tree was inferred by the neighbor-joining method using the Poisson correction. The robustness of the tree was evaluated by the non-parametric bootstrap procedure implemented in SeaView (1,000 replicates of the original dataset).

A local database gathering 2775 prokaryotic complete proteomes available in November 2014 at the NCBI (http://www.ncbi.nlm.nih.gov/) was built and formatted with the FORMATDB program of the BLAST package (version 2.2.26) [23]. This database contained archaeal and bacterial proteomes, including that of three *Azospirillum* strains (*A. brasilense* Sp245, *A. lipoferum* 4B and *Azospirillum* sp. B510). The proteome of *A. brasilense* CBG497 (6,185 protein sequences) was retrieved from MaGe [16] and added to the local database. The protein sequences of the 49 HK and the 62 REC domains contained in the 49 HyHKs from *A. lipoferum* 4B were used as queries for similarity search. For each domain sequence, the 100 most similar homologues were identified using BLASTP, with the default parameters, except the filter on low complexity regions that was turned off. The sequences were retrieved and the corresponding 111 datasets were aligned with MAFFT v7.123b [24] with the L-INS-i option. The resulting multiple alignments were trimmed with the Gblocks program [21] implemented in the SeaView 4.5.4 software [22], allowing smaller final blocks and less strict flanking positions. Maximum likelihood (ML) phylogenetic trees corresponding to the 111 cleaned alignments were inferred with PHYML-3.1 [25]. We used the Le and Gascuel model [26], with a gamma distribution in order to take into account evolutionary rate variations across sites (4 relative substitution rates), and the NNI + SPR topology search strategy. The alpha parameter of the gamma distribution was estimated with PHYML. The robustness of the branches of the resulting ML trees was estimated with the Shimodaira-Hasegawa-like procedure implemented in PHYML. A similar procedure was used to infer the phylogeny of the 49 and that of the 62 *A. lipoferum* HK and REC domains, respectively.

## Results & discussion

### Enrichment of TCS genes in *Azospirillum* compared to other plant-associated bacteria

General features of *Azospirillum* TCS proteins retrieved from the P2CS database and the Mage platform are summarized in Table 1. The complete genomes chosen for this analysis include those of: (i) *Azospirillum* sp. B510, a strain isolated from disinfected rice stems in Japan [27], (ii) *A. lipoferum* 4B isolated from rice in France, (iii) *A. brasilense* Sp245 isolated from wheat in Brazil [14], and (iv) *A. brasilense* CBG497, isolated from maize grown on an alkaline soil (pH 8) in Mexico [15]. First, *Azospirillum* devotes between 3.3 % (*A. brasilense* Sp245) to 3.9 % (*A. lipoferum* 4B) of its genes to TCS ORFs. Indeed, *A. lipoferum* 4B possesses a total of 244 putative TCS genes out of 6,233 ORFs, comprising 118 genes encoding HKs (69 classic HKs and 49 HyHKs) and 121 RRs. *Azospirillum* sp. B510, the closest relative of *A. lipoferum* (Additional file 2: Figure S1), has 260 TCS genes, 122 HKs (75 classic HKs and 47 HyHKs) and 132 RRs. Moreover, no phosphotransfer protein (*i.e.* stand-alone HisKA or Hpt domain-containing protein) has been detected in *A. lipoferum* 4B and in *Azospirillum* sp. B510 genomes. However Hpt domain detection has often been hampered by low sequence conservation, thus, *Azospirillum* genomes may encode more proteins containing Hpt domains than detected in our study [28]. *A. brasilense* Sp245 possesses a total of 259 TCS genes, 129 HKs (74 classic HKs and 55 HyHKs), 124 RRs and 2 phosphotransfer proteins (2 Hpt). Finally, *A. brasilense* CBG497 has a total of 227 putative TCS genes encompassing 110 HKs (60 classic HKs and 50 HyHKs), 111 RRs and two phosphotransfer proteins (1 HisKA and 1 Hpt). CheA gene numbers retrieved in this study are in accordance with previous *Azospirillum* whole-genome analyses [14, 15]. As the Che systems have been previously described elsewhere, we chose to specifically focus on TCS proteins that are not part of Che-like systems. Analysis of the receiver (REC-containing domains RRs and HyHKs): transmitter (HisKA and HATPase-containing domains, HKs and HyHKs) values revealed a ratio closed to 1.4, thus suggesting that one HK may phosphorylate more than one RR.

**Table 1:** General features of *Azospirillum* TCS genes

Strains and feature	TCS type	Chromosome	p1	p2	p3	p4	p5^a^	p6	Total^b^
*A. lipoferum* 4B									
Sensing HK	Classic HK	34	8	11	6	4	6	-	69
	Hybrid HK	18	5	5	3	11	5	2	49
	CheA	2	-	1	-	2	-	-	5
Output response	RR	61	9	18	9	15	9	-	121
Phosphotransfer	HisKA	-	-	-	-	-	-	-	-
	Hpt	-	-	-	-	-	-	-	-
Total		115	22	35	18	32	20	2	**244**
ORF		2904	883	640	555	599	415	237	6233
Size (Mpb)		2.99	1.04	0.75	0.65	0.65	0.48	0.29	**6.85**
*Azospirillum* sp. B510									
Sensing HK	Classic HK	31	13	6	10	4	11	-	75
	HyHK	22	9	1	3	8	4	-	47
	CheA	2	2	-	-	2	-	-	6
Output response	RR	60	23	7	13	15	14	-	132
Phosphotransfer	HisKA	-	-	-	-	-	-	-	-
	Hpt	-	-	-	-	-	-	-	-
Total		115	47	14	26	29	29		**260**
ORF		3287	1263	693	589	598	464	232	7126
Size (Mpb)		3.31	1.46	0.72	0.68	0.63	0.54	0.26	**7.60**
*A. brasilense* Sp245									
Sensing HK	Classic HK	31	22	8	5	7	1	-	74
	HyHK	19	13	6	9	7	1	-	55
	CheA	1	2	1	-	-	-	-	4
Output response	RR	47	37	19	9	11	1	-	124
Phosphotransfer	HisKA	-	-	-	-	-	-	-	0
	Hpt	1	1	-	-	-	-	-	2
Total		99	75	34	23	25	3	-	**259**
ORF		3309	1812	922	824	691	163	125	7846
Size (Mpb)		3.02	1.77	0.91	0.78	0.69	0.19	0.17	**7.53**
*A. brasilense* CBG497									
Sensing HK	Classic HK	26	17	9	4	4	-	-	60
	HyHK	21	14	7	2	6	-	-	50
	CheA	1	2	1	-	-	-	-	4
Output response	RR	47	32	20	5	7	-	-	111
Phosphotransfer	HisKA	1	-	-	-	-	-	-	1
	Hpt	-	1	-	-	-	-	-	1
Total		96	66	37	11	17	-	-	**227**
ORF		2895	1430	643	512	583	-	122	6185
Size (Mpb)		2.90	1.60	0.73	0.49	0.60	-	0.15	**6.47**

^a^Absent in *A. brasilense* CBG497

^b^For each strain, the total number of TCS genes and genome size are highlighted in bold

The number of TCS per genome is positively correlated with genome size [5, 6]. This number appears to correlate strongly with ecological niche, as bacteria living in relatively constant environments tend to have few TCS and contrarily, free-living bacteria experiencing fluctuating environmental conditions, or having complex physiological behaviours tend to be enriched in TCS genes [29, 30]. Thus, to provide a suitable context for our comparisons, we selected 85 complete prokaryotic genomes from bacteria living in close association with plants and experiencing contrasting plant-associated lifestyles (*i.e.* 34 free-living bacteria or PGPR, 25 symbionts, 26 plant pathogens) and compared their TCS gene repertoire with those retrieved in *Azospirillum* (Additional file 3: Table S2**;** Additional file 4: Table S3; Additional file 5: Table S4 and Additional file 6: Table S5). We also included eight other genomes in our dataset, *i.e.* those of: (i) the two model bacteria, *Escherichia coli* K-12 and *Bacillus subtilis* subsp. *subtilis* str. 168*,* whose TCS have been extensively described [28, 31]; (ii) the delta-proteobacterium *Myxococcus xanthus* DK 1622 as a representative of large genome size organism (9.14 Mpb), characterized by a complex social behaviour (fruiting body formation) and described as containing a very large number of TCS genes (*i.e.* 278) [18]; (iii) *Magnetospirillum magneticum*, *Rhodospirillum centenum*, *R. photometricum* and *R. rubrum*, free-living aquatic members of the *Rhodospirillaceae* family that are the closest *Azospirillum* relatives, as TCS gene content is also influenced by bacterial phylogeny [14, 32].

**Fig. 1:**
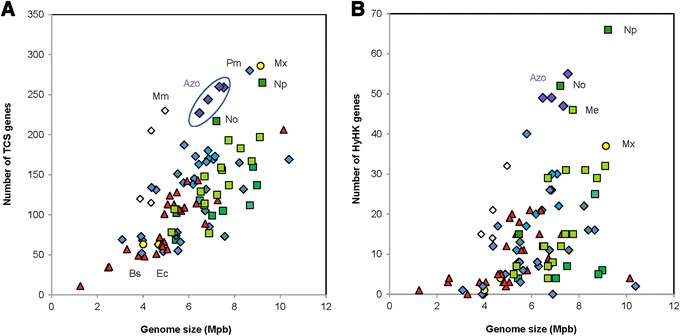
Correlation between the number of TCS genes and genome size among plant-associated bacteria. **a** Number of all TCS genes. **b** Number of genes encoding HyHKs. Symbols represent, yellow circles: model organisms (*B. subtilis*, *E. coli*, *M. xanthus*); red triangles: phytopathogens; green squares: plant symbionts (light green for Rhizobia, medium green for *Frankia* and dark green for *Nostoc*); blue diamonds: PGPR; open diamonds: aquatic *Rhodospirillaceae*. Abbreviations are, Azo: *Azospirillum*; Bs: *B. subtilis* BSn5; Ec: *E. coli* K12; Me: *Methylobacterium* sp. 4–46; Mm: *M. magneticum* AMB-1; Mx: *M. xanthus* DK1622; No: *Nostoc* sp. PCC 7120; Np: *N. punctiforme* PCC 73102; Pm: *Paenibacillus mucilaginosus* KNP414

Our analysis demonstrated a positive correlation between TCS total gene number and genome size, which is in accordance with previous studies (Fig. 1a) [5, 6]. More importantly, compared to other plant-associated bacteria with similar genome size, all four *Azospirillum* strains appeared to be enriched in TCS. Indeed, the genomes of the beneficial biocontrol fluorescent *Pseudomonas protegens* CHA0 (6.9 Mbp) and *P. fluorescens* SBW25 (7.1 Mbp) encode respectively 170 and 173 TCS genes, representing 2.8 % and 2.9 % of their ORFs; genomes of the nitrogen fixing symbionts of legumes, *Rhizobium tropici* CIAT 889 (6.7 Mpb), *R. leguminosarum* bv. *trifolii* WSM 1325 (7.4 Mpb) and *Cupriavidus taiwanensis* LMG19424 (6.5 Mpb), encode only respectively 112, 160 and 146 TCS genes (Fig. 1a; Additional file 1: Table S1). The only organisms displaying a number of TCS genes larger than that of *Azospirillum* are organisms with larger genomes. These organisms comprise the filamentous cyanobacterium *Nostoc punctiforme* PCC 73102 (9.2 Mp encoding 286 TCS genes) and *M. xanthus* DK 1622 (9.1 Mpb encoding 286 TCS genes), two bacteria capable of complex behaviors, such as cell differentiation and multicellular development (respectively heterocysts and fruiting bodies formation) [5, 18, 33]. Additionally, the nitrogen-fixing firmicute *Paenibacillus mucilaginosus* KNP414 was also found to possess many TCSs (8.6 Mpb encoding 280 TCS). This feature has previously been unveiled in one of its closest relatives *Paenibacillus vortex*, in which enrichment in TCS presumably correlated with its sophisticated social motility [34]. Although *Azospirillum* displays no complex cellular behaviours such as those described above, transformation of vegetative cells to desiccation-resistant encysting forms under limiting cultural conditions has been reported [35] and this process may necessitate the integration of a multitude of signals. As for the other *Rhodospirillaceae*, the magnetotactic alpha-proteobacterium *M. magnetotaticum* AMB-1 showed an unexpectedly high number of TCS, as high as that of *Azospirillum* despite a smaller genome (4.9 Mpb encoding 230 TCS genes) whereas the genome of *Rhodospirillum centenum* CW (4.3 Mpb) encodes only 115 TCS. The presence of numerous signal transduction genes was shown to be a common feature in magnetotactic bacteria, enabling them to tightly adjust their physiology to adapt to varying environmental conditions [36]. This suggests that most TCS genes among the *Rhodospirillaceae* family have been acquired prior to speciation and thus lineage specific gene loss occurred among the *Rhodospirillum* genus (Additional file 2: Figure S1). However, our phylogenetic analysis of HyHK of *A. lipoferum* 4B (see below) contradicts this hypothesis.

### Abundance of orphaned and complex TCS genes in *Azospirillum* genomes

TCS genetic organization is important information regarding potential TCS partnership as many genes encoded within the same operon function in the same pathway. In this regard, we classified *Azospirillum* TCS genes according to their genetic context as either (i) orphan when no TCS gene was found adjacent to another TCS gene, (ii) paired when two TCS genes, whatever their nature (HK or RR), were found adjacent and (iii) complex when more than two genes were contiguous [37]. The proportion of *Azospirillum* TCS genes that are paired varies from 36–43 %, whereas orphaned TCS genes account for 38–52 % of TCS genes (Table 2). Besides, 15–18 % of *Azospirillum* TCS genes are organized in complex clusters. Nonetheless, these complex loci organization vary among the four strains, in terms of number and combination. The smallest replicon of *A. lipoferum* 4B (p6, 237 ORFs), the and only replicon that has the features of typical plasmids [14], harbours only two genes encoding orphan HyHK (AZOLI_p60219 and AZOLI_p60221) that display no orthologues in other *Azospirillum* genomes. Still in *A. lipoferum* 4B a hexad of TCS chromosomally-encoded genes is particularly intriguing, spanning from AZOLI_0973 to AZOLI_0978, with two classic HKs followed by four RRs (Additional file 3: Table S2). However, the putative phosphotransfer route deduced from this configuration is difficult to understand and would need further experimental investigation.

**Table 2:** Genetic organization of *Azospirillum* TCS

Strain	Orphan	Pair	Triad	Tetrad	Pentad	Hexad	TOTAL
*A. lipoferum* 4B	106	48	5	4	1	1	244
*Azospirillum* sp. B510	100	56	12	3	0	0	260
*A. brasilense* Sp245	128	46	10	1	1	0	259
*A. brasilense* CBG497	101	42	10	3	0	0	227

In comparison, the majority of *E. coli* TCS were organized as pairs (71.5 %), while orphaned and complex TCS accounted for respectively 14.5 % and 14.5 % [28]. In *M. xanthus*, only 29 % TCS genes were found to be adjacent to each other, 33 % of them formed complex clusters and 38 % were categorized as orphans, a repartition resembling that found in *Azospirillum* [18]. Strikingly, a high number of *M. magnetotaticum* TCS genes were classified as orphans (63 %), while only 20 % were found to lie adjacent to another TCS gene, and 17 % were clustered in complex loci (data not shown).

The enrichment of non-conventional TCS genetic organization has been observed in bacterial species that have large genomes, thus supporting the idea that as genome size increases, communication among TCS proteins may be favored leading to complex signal transduction pathways [37, 38]. Altogether, this suggests that *Azospirillum* have evolved complex signalling transduction pathways that may allow a better adaptation to a given niche.

### Overrepresentation of HyHKs in *Azospirillum*

As *Azospirillum* strains appeared to be enriched in TCS compared to other plant-associated bacteria of similar genome size, we next focused on HKs and RRs separately. For these two sets of proteins, the same general trend between abundance and genome size can be observed, with *Azospirillum* displaying elevated numbers of HKs and RRs for its genome size (Fig. 1b and data not shown). Notwhistanding this observation, the relationship between the numbers of encoded HyHKs within our set of selected genomes of plant-associated bacteria, did not follow a linear relationship between these two parameters (Fig. 1b), as previously reported [39]. Surprisingly, *Azospirillum* genomes were found to contain a high number of HyHKs genes (49 to 55) compared to other plant-associated bacteria of similar genome size: *P. protegens* CHA0 (30 HyHKs)*, P. fluorescens* SBW25 (22 HyHK*s*)*, R. tropici* CIAT 899 (4 HyHKs*), R. leguminosarum* bv. *trifolii* W*SM* 1325 (16 HyHKs) and *C. taiwanensis* LMG19424 (12 HyHKs). Even more stunning is the fact that the number of HyHKs in *Azospirillum* overtakes the numbers found in bacteria with larger genomes (37 in *M. xanthus* DK 1622; 16 in *P. mucilaginosus* KNP414), with the only exception of two cyanobacterial strains (52 in *Nostoc* sp. PCC 7120 and 66 in *N. punctiforme* PCC 73102). HyHKs sensing proteins are involved in phosphorelays signalling cascades that may offer additional regulatory checkpoints allowing the fine-tuning of some physiological behaviours in response to given signals. Further evidence now supports some additional roles of HyHKs, such as mediating direct protein-protein interactions [40]. Therefore, HyHKs may play a pivotal role for rapid acclimation and fine-tuning of *Azospirillum* physiology in the soil and root environments.

**Fig. 2:**
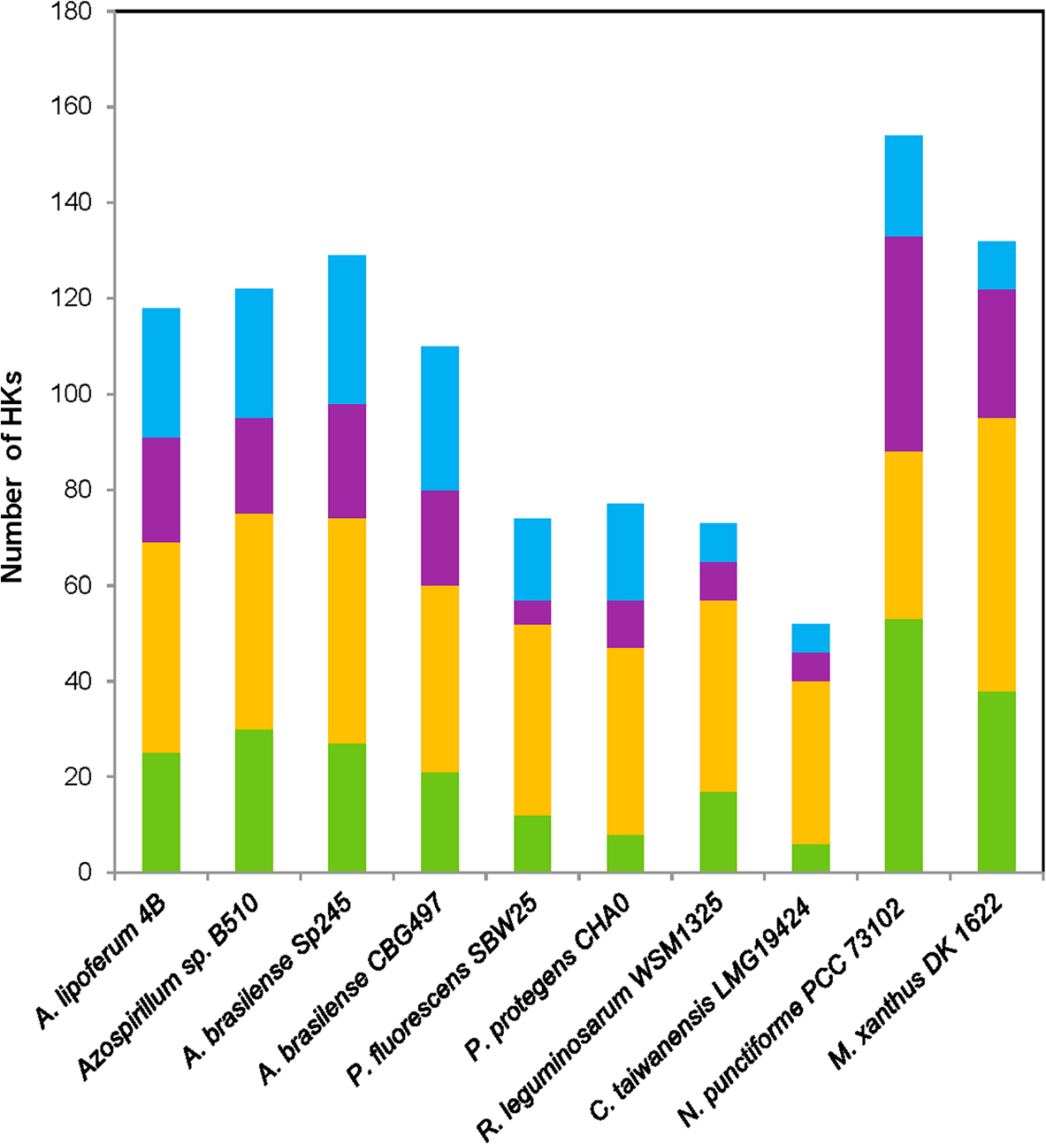
Predicted subcellular localization of HKs from *Azospirillum* and other plant-associated bacteria. For each selected strain, the predicted subcellular localization of HKs was retrieved from the P2CS database or using the TMHMM server v. 2.0 for *A. brasilense* CBG497 HKs. Green: soluble classic HKs; orange: membrane classic HKs; purple: soluble HyHKs; blue: membrane HyHKs

The cellular localization of each HK was further assessed by the presence or absence of transmembrane helices (Additional 3: Table S2; Additional file 4: Table S3; Additional file 5: Table S4; and Additional file 6: Table S5). A mean of 39 % (±1 %) of total *Azospirillum* HKs were predicted to contain no transmembrane segment, suggesting a cytosolic subcellular localization (Fig. 2). Previous genome analyses predicted that only 12–27 % of HKs have no transmembrane regions, the maximum being reported for cyanobacteria and myxobacteria (50 % *M. xanthus* and 64 % *N. punctiforme* of cytosolic HKs) [5, 19]. In other plant-associated bacteria, the proportion of soluble HKs were respectively 23, 23, 34 % and 23 % in *P. protegens* CHA0, *P. fluorescens* SBW25, *Rhizobium leguminosarum* bv. trifolii WSM1325 and in *C. taiwanensis* LMG 19424 strains (Fig. 2). By comparing the proportion of potentially soluble and membrane HKs between classic HKs and HyHKs in *Azospirillum* genomes, a slightly higher proportion of HyHKs were found to be soluble (mean of 42 % ± 1 %) compared to their classical counterparts (mean of 37 % ± 1 %), although not statistically significant (Fig. 2). Soluble sensing HKs were initially thought to sense intracellular cues (ATP, redox potential, reactive oxygen species) allowing the bacteria to monitor the internal metabolic status of a cell. However, membrane-diffusible signals could also be perceived by soluble HKs; consequently these systems could be relevant in the complex rhizosphere ecosystem, allowing the integration of a wide array of physicochemical parameters and plant-exuded compounds. Furthermore, the prevalence of cytosolic HyHKs within an organism could provide a selective advantage by favouring spatial proximity with its cognate RR, therefore increasing the velocity and efficiency of the phosphotransfer signalling route [41].

### Insight into domain architecture of *Azospirillum* HKs

#### Sensing domains

Analysis of sensing domains is crucial to understand the nature of the signals detected by individual HKs. A search for common and uncommon input domains described previously [42] was achieved among *Azospirillum* HKs. Intriguingly, HKs without any input domain represent around 28 % (±0.35 %), thus suggesting that they might contain either unknown domains, or that they may be part of more complex signalling systems, acting in concert with auxiliary proteins (Table 3; Additional 3: Table S2; Additional file 4: Table S3; Additional file 5: Table S4; and Additional file 6: Table S5) [43]. For HKs containing an input domain, a mean of 13 (±1.5) distinct sensing domains were identified among *Azospirillum* HKs, with *A. lipoferum* 4B and *Azospirillum* sp. B510 exhibiting a wider diversity of input domains than the *A. brasilense* strains (Table 3). By comparison, plant-associated bacteria with similar genome size contained a slightly lower diversity of input domains, from 9 (*R. tropici* CIAT899) to 12 (*P. protegens* CHA0). Finally, *M. xanthus* DK 1622 and *N. punctiforme* PCC 73102 were found to have respectively 14 and 11 distinct sensing domains among their HKs (data not shown).

**Table 3:** Input domains found in *Azospirillum* sensing HKs

Strains	*A. lipoferum* 4B		*Azospirillum* sp. B510		*A. brasilense* Sp245		*A. brasilense* CBG497		Putative signal(s) detected
HK type^a^	ClaHK	HyHK	ClaHK	HyHK	ClaHK	HyHK	ClaHK	HyHK	
Abundant input domains									
PAS / PAC	43	42 / 1	35	35 / 3	46	71	35	71	Small molecules, ions, gases, light, and redox state sensing
HAMP	12	18	18	9	14	11	11	13	Signal transduction
Uncommon input domains									
GAF	3	4	3	4	7	4	5	4	Redox or oxygen sensing; cGMP binding
PHY	1	-	1	1	1	1	1	1	Tetrapyrroles; light-sensing
CHASE	3	-	2	-	3	-	2	-	Small molecules recognition
MASE	1	1	2	-	1	-	1	1	Membrane associated sensor
2CSK_N	1	-	3	-	1	-	1	-	Unknown; commonly found in N-terminal parts of HK
Cache	-	4	-	4	2	3	-	-	Small molecules recognition
MHYT	-	2	-	3	-	-	-	2	Putatively involved in metal sensing
cNMP_binding	1	-	1	-	-	-	-	-	Cyclic nucleotide monophosphate-binding
S_TKc	1	-	1	-	-	-	-	-	Serine/Threonine kinase catalytic domain
SBP_bac_1	1	-	1	-	-	-	1	-	Bacterial extracellular solute binding protein
CheB methylesterase	-	-	1	2	-	-	-	-	Chemotaxis
MeTRC	-	-	1	2	-	-	-	-	Methyl transferase
NIT	-	-	1	-	-	-	-	-	Nitrate and nitrite responsive
PBPb	-	-	-	-	1	-	1	-	High-affinity periplasmic solute-binding protein

^a^ *ClaHK* Classic Histidine Kinase, *HyHK* Hybrid Histidine Kinase

A vast majority of input domain-containing HKs were found to contain a PAS (Per-Arnt-Sim) domain (Table 3; Additional 3: Table S2; Additional file 4: Table S3; Additional file 5: Table S4; and Additional file 6: Table S5) [44]. PAS domains have been shown to sense a wide array of intracellular and/or extracellular signals, such as small molecules, ions, light, gases, redox state [45]. PAS domains were found to be more abundant among HyHKs than among classic HKs; indeed, 43 PAS domains were distributed over 69 classic HKs whereas 42 of them were distributed over 49 HyHKs (Table 3). The HAMP domain (Histidine kinases, Adenylyl cyclases, Methyl binding proteins, Phosphatases) was found in 31 % (±2 %) of input domain-containing HKs. This domain converts signals from periplasmic sensor domains to cytoplasmic output domains via conformational changes [46]. The GAF domain (cGMP-specific phosphodiesterases, Adenylyl cyclases and FhlA) was found in all *Azospirillum* strains but to a lesser extent (10 % ± 1 % of input domain-containing HKs). All four *Azospirillum* strains contain at least one photoreceptor PHY domain, displaying the *N*-terminal domain arrangement reminiscent of bacterial phytochromes (PAS-GAF-PHY) (Table 3) [47]. The classical HK containing this PHY domain is a type 1 bacteriophytochrome (AZOLI_p20424, AZL_a05830, AZOBR_p440075 and AZCBG_p440017). In *A. brasilense* Sp7, this protein was shown to be involved in tolerating red light mediated photodynamic stress, and was further shown to be involved in the regulation of swarming motility [48].

Some sensing domains were found to be absent in the two *A. brasilense* strains but present in *A. lipoferum* 4B and *Azospirillum* sp. B510, such as cNMP binding domain (PF00027) and S_TK (PF00069), that are respectively involved in cyclic nucleotide monophosphate binding detection and crosstalk between Ser/Thr signalling process. Conversely, one PBPb (Periplasmic solute-Binding Protein) domain (PF12974) was found associated only with the two *A. brasilense* strains (AZOBR_p230031 and AZCBG_p2110061). Bacterial periplasmic solute-binding proteins often function as receptors regulating chemotaxis, transport systems and/or signal transduction pathways [49]. Furthermore, some sensing domains were found exclusively in association with a particular *Azospirillum* strain. This is the case for the NIT (Nitrate- and nitrite- sensing) domain (PF08376) that was found only in a single *Azospirillum* sp. B510 classic HK (AZL_012680). Its association with two other PAS domains suggests that it could detect change in nitrate or nitrite concentration [50]. However, the exact roles of these sensing domains are difficult to apprehend, as for most functionally characterized HKs, the signal(s) detected are still unknown.

Altogether, it seems that *Azospirillum* strains have evolved a multitude of sensing strategies that may favor adaptation to particular ecological niches.

#### Architectures of *Azospirillum* HK

Domain architecture has been proven to be very informative regarding the putative function of signalling proteins [51]. Analysis of HK domain arrangement in *Azospirillum* showed a wide array of domain combinations among HyHKs compared to their classical counterparts (Table 4; Additional 3: Table S2; Additional file 4: Table S3; Additional file 5: Table S4; and Additional file 6: Table S5). Indeed, when HKs were classified according to the number and order of their input domains (whatever the nature of the sensing domain), transmitter (comprising HisKA and HATPase domains) and receiver domains, only six distinct domain architecture combinations could be found among *Azospirillum* classical HKs (data not shown). On the contrary, a remarkable diversity of domain architectures was observed when considering HyHKs (respectively 19, 19, 16, 13 distinct domain architectures in *A. lipoferum* 4B, *Azospirillum* sp. B510, *A. brasilense* Sp245 and CBG497) (Table 4). Firstly, HyHKs composed of only one transmitter and one receiver domain constitute the majority of HyHK domain architectures found in *Azospirillum* genomes (67 ± 2 %), whereas the remaining 33 % of HyHKs exhibit atypical domain architectures with multiple transmitter and receiver domains. Such complex domain architectures are puzzling as they may give rise to complex phosphotransfer pathways within each protein. Secondly, while around 25 % of HyHKs consist solely of transmitter and receiver domains without any input domain, the majority of HyHKs seem to have accumulated multiple sensing domains, with up to 10 sensing domains (including nine tandem repeated HAMP-GAF) in two *A. lipoferum* 4B and *Azospirillum* sp. B510 orphan HyHKs (AZOLI_p30171, AZL_c01470).

**Table 4:**
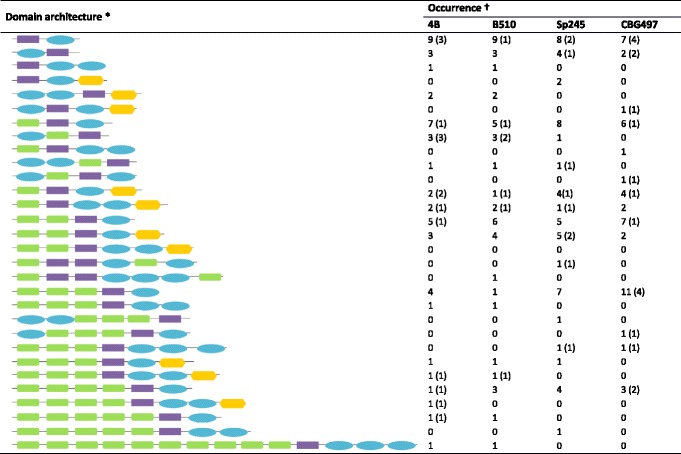
Schematic representation of HK domain architecture and occurrence among *Azospirillum* genomes

*Domain architecture was classified according to the nature and number of input (green) transmitter (purple), receiver (blue) and Hpt (orange) domains

†Numbers in brackets indicate HyHK that are found associated with a cognate RR

The existence of multi-domain proteins harbouring several sensing domains suggests their ability to detect a wider range of signals. This was functionally validated by deleting the PAS1 or the PAS2 domains of ArcS of *Shewanella oineidensis,* a HyHK comprising one extracellular Cache domain, two PAS domains, a transmitter domain and two REC domains [52]; deletion of either the PAS1 or the PAS2 domain did not result in the same mutant phenotype suggesting that signal detection occurs in different ways. The two REC domains may have different roles regarding phosphotransfer route as revealed by *in vitro* phosphotransfer studies [52]. Altogether, *Azospirillum* may have adapted to their environment by retaining new combinations of existing domains, a process that eventually contributed to increase the genetic diversity of HyHKs.

### Evolutionary origin of *Azospirillum* HyHKs

**Fig. 3:**
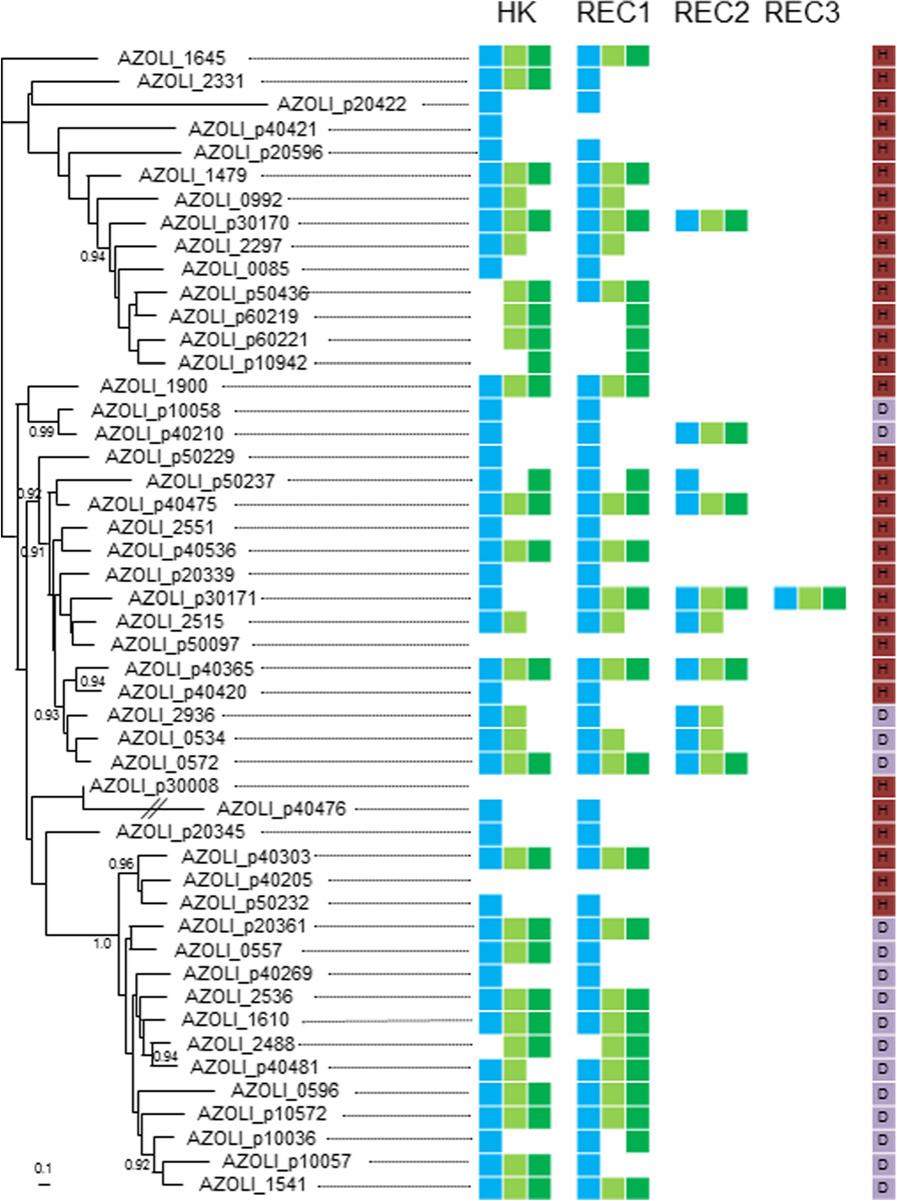
Evolutionary origin of 49 *A. lipoferum* 4B HyHKs. The phylogenetic tree showed the maximum likelihood relationships among the 49 transmitter domains (49 sequences, 265 positions). Numbers at branch correspond to SH-like supports calculated with PHYML (for clarity values lesser than 0.90 are not shown). The scale bar indicates the average number of substitution per site. The colored squares indicate the presence of orthologous transmitter (HK) and REC domains in *Azospirillum* sp. B510 (*blue*), *A. brasilense* CBG497 (*light green*) and *A. brasilense* Sp245 (*dark green*). According to phylogenetic analyses, the probable origin of the *A. lipoferum* 4B HyHK is indicated (*red, horizontal gene transfer; purple, gene duplication*)

Such diverse combinations of domains in addition with the large number of HyHKs found in *Azospirillum* genomes prompted us to investigate how this expansion of HyHKs arose. To this end, phylogenetic analysis of the 49 transmitter and 62 REC domains from the 49 HyHKs of *A. lipoferum* 4B (*i.e.* a total of 111 domains) has been performed (Fig. 3). First, we showed that transmitter and REC domains found in three *A. lipoferum* 4B HyHK proteins have no orthologue in the three others *Azospirillum* strains, indicating that these proteins are specific to *A. lipoferum* 4B and may have originated recently in this genus. In contrast, we showed that 33 HyHKs display orthologous transmitter and REC domains, meaning that the association between these domains is more ancient in the *Azospirillum* genus. More precisely, 15 orthologous HyHKs are found in the four *Azopirillum* genomes, and 12 are common to *A. lipoferum* 4B and *Azospirillum* sp. B510, which is in accordance with their phylogenetic relationship (Additional file 2: Figure S1). Finally, 13 HyHKs displays domains with different evolutionary origins.

Phylogenies of the transmitter and the REC domains suggested that the expansion of these HyHKs has been driven by horizontal gene transfer (HGT, Fig. 3). In fact, 32 out of 49 transmitter domains were clustered into clades containing sequences of various bacteria that are not related to *Azospirillum* (not shown). Although phylogenies of the REC domains were overall less resolved, due to the low number of conserved positions for phylogenetic reconstruction, the same trend could be observed. A clear illustration of such HGT lies in AZOLI_p30008, one HyHK specific to the *A. lipoferum* species (Fig. 3); indeed, both transmitter and REC domains of AZOLI_p30008 (YP_005040549.1) clustered with several HKs of *Methylobacterium* (Additional file 2: Figure S2A and S2B). *Azospirillum* spp. are known for their propensity for genome plasticity [53, 54] and whole-genome analysis revealed that *Azospirillum* underwent extensive HGT allowing the transition from aquatic to terrestrial environments and association with plants [14].

Duplication events also played an important role in the expansion of HyHKs, either at the gene or at the domain level. As such, AZOLI_p10058 (YP_004973569.1) and AZOLI_p40210 (YP_004975186.1) likely originated from the duplication of an ancestral gene that was present in the common ancestor shared by *A. lipoferum* 4B and *Azospirillum* sp B510 (Fig. 3, Additional file 2: Figure S3A and S3B). Interestingly, the second REC domain found in AZOLI_p40210 has a different origin, being present in the four *Azospirillum* strains (Fig. 3). This suggested that a domain rearrangement occurred after the duplication event.

Some HyHKs may have also arisen through duplication of a classical HK and fusion with a REC domain of a RR, as previously described [38, 39]; such a scenario has likely occurred for AZOLI_1645, in which the transmitter domain clusters with those of two classical HKs (YP_003448836.1 and YP_005031706.1) and the REC domain grouping with that of a RR (YP_003452077.1) (data not shown). Finally, some HyHKs could have resulted from more complex scenarios involving both HGT and duplication events, as in the case of the transmitter domain and the two REC domains of AZOLI_2936 (data not shown).

In summary, the evolutionary history of HyHKs in *A. lipoferum* 4B is complex, involving HGT (mainly from proteobacteria), duplications events as well as recombination events allowing domain shuffling. Domain shuffling often goes hand in hand with lineage specific extension (LSE), and LSE genes are much more likely to be present as orphans as depicted above [30]. Genome-wide phylogenetic distribution analysis of HKs across 207 prokaryotic genomes revealed than more than 70 % of recent duplications displayed an input domain structure different from that of their closest paralogue [30]. *Azospirillum* HyHKs might have evolved by lateral recruitment of additional sensing domains, thus enabling the detection of a large panel of signals [39]. Altogether, this suggests that the expansion of the HyHK family in *Azospirillum* may have arisen in response to unique challenges that members of this genus faced.

### Diversity of output domains of *Azospirillum* RRs

Contrarily to HK transmitter domains, classification of RRs based on sequence similarity cannot help fully determine a putative biological function. However, classification of RRs based on output domain and architecture has been proven to be useful to decipher putative RR functions [55]. Therefore, *Azospirillum* RRs were classified according to their output domains as shown in Table 5. Firstly, CheY-like RRs containing standalone REC domains and lacking an output domain were found to be overrepresented among *Azospirillum* TCS (mean of 32 genes, *i.e.* 38.7 ± 1.2 % of total RRs). In comparison, genes encoding RRs consisting of solely a REC domain represent respectively 10.1 % and 13.8 % of *P. protegens* CHA0 and *P. fluorescens* SBW25 total RRs (data not shown). Those genes represent nearly one third of total RRs encoded within the *R. leguminosarum bv. trifolii* WSM 1325 genome, while they represent 23.3 % of *R. tropici* CIAT 899 total RRs. Intriguingly, overrepresentation of RRs with standalone REC domains was found in *N. punctiforme* and *M. xanthus* that respectively represent 36 % and 34 % of the total of their RRs [18, 33]. CheY-type RRs was first described in chemotaxis regulation in *E. coli*, but their roles go far beyond as they can also act as connectors between TCS partners thereby facilitating crosstalk, feedback, and phosphorelays within the two-component phosphorylation network [56]. Interestingly, only *A. brasilense* Sp245 genome harbours one gene encoding a FrzZ-like RR (AZOBR_p330125), that consists of two REC domains arranged in tandem. In *M. xanthus,* FrzZ was shown to act as a major RR of the chemotaxis system, controlling cell motility and fruiting body formation [57]. Surprisingly, our previous transcriptional analyses of *A. lipoferum* 4B interacting with rice roots revealed that CheY-like standalone RRs were all differentially expressed, strongly suggesting that they may be involved in *Azospirillum*-plant interaction [58]. However, the exact role played by these single domain RRs needs further investigation.

**Table 5:** Output domains of *Azospirillum* RRs

Output domain	RR family	*A. lipoferum* 4B	*Azospirillum* sp. B510	*A. brasilense* Sp245	*A. brasilense* CBG497
Stand-alone REC	CheY	39	37	42	36
	FrzZ	-	-	1	-
DNA-binding	NarL	22	21	22	16
	NtrC	8	10	8	8
	OmpR	19	27	19	16
	LytTR	2	2	1	1
	PrrA	2	2	3	3
	MarR	-	1	2	-
Chemotaxis	CheB	5	6	4	4
	CheY	5	6	4	4
	CheV	-	-	-	-
RNA-binding	AmiR-NasR	-	-	-	-
c-di-GMP signalling	RpfG	5	4	3	4
	VieA	-	-	-	-
	PleD	7	5	6	5
	PleD-VieA	1	1	0	-
Ser/Thr phosphorylation	RsbU	4	3	4	4
Unknown	Unclassified	2	7	5	10
	Total	121	132	124	111

While most of *Azospirillum* RRs with known output domains (64 % ± 1 %) contain a DNA-binding domain (39 % ± 2 %) and may serve as transcriptional regulators, no RNA-binding domain was detected among *Azospirillum* RRs, suggesting that no control of gene expression through antitermination is mediated by RRs. Furthermore, enzymatic output domains are common among RRs as 17 % (±1) of them are involved in the modulation of the secondary messenger c-di-GMP or Ser/Thr phosphorylation, suggesting interconnection of *Azospirillum* TCS with other signal transduction pathways.

### Evolutionary conserved TCS and accessory TCS

**Fig. 4:**
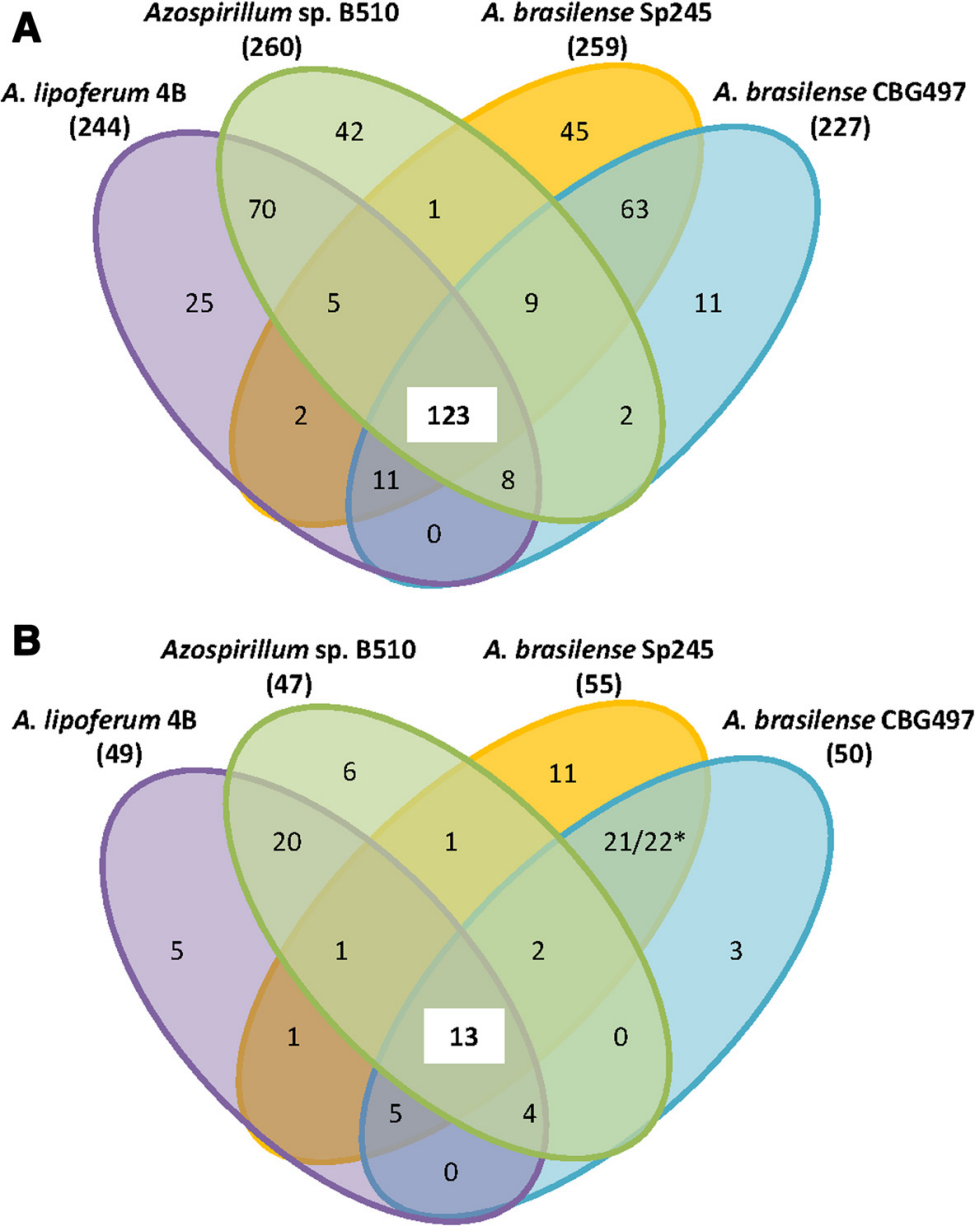
Venn diagrams representing numbers of orthologous and unique TCS genes between and among the four *Azospirillum* strains studied. **a** Numbers of orthologous total TCS genes; **b** Numbers of orthologous genes encoding HyHKs. The number of orthologous TCS genes was determined by reciprocal best blast hit (amino acid identity percentage greater than 35 % and query coverage greater than 75 %). The total number of TCS coding genes within each genome is listed in brackets next to the strain name. * Among HyHKs common to both *A. brasilense* strains, 21 were classified as HyHKs in *A. brasilense* Sp245 and 22 in *A. brasilense* CBG497

The most probable set of orthologous TCS proteins shared by the four *Azospirillum* strains was identified based on a reciprocal best BLAST hit criterion, as previously described [15]. A total of 123 putative TCS proteins were found to be common to all genomes, thus representing half of *Azospirillum* TCS gene content (Fig. 4; Additional 3: Table S2; Additional file 4: Table S3; Additional file 5: Table S4; and Additional file 6: Table S5). This core set of *Azospirillum* TCS comprises 36 HKs, 13 HyHKs and 71 RRs (Fig. 4). Among the orthologous TCS genes, 29 TCS pairs were shared by the four *Azospirillum* strains, including 19 pairs displaying synteny over at least five genes, therefore reflecting possible co-evolution between these cognate HKs and RRs [29, 38]. These TCS may play important roles in regulating general functions common to all strains such as cell growth mechanisms and/or survival in the soil and the rhizosphere ecosystems, and a few of them are discussed hereafter (Table 6). A tetrad of TCS genes is conserved among the four *Azospirillum* strains and encodes the global nitrogen regulatory system NtrBC and NtrXY, shown to control the transcription of genes involved in nitrogen fixation and assimilation under nitrogen-limitation (Table 6) [59, 60]. The KdpD/KdpE system is also common to all strains; in *Sinorhizobium meliloti*, this system controls the expression of the *kdpABC* operon encoding a potassium uptake pump and was shown to play a key role in potassium osmoadaptation and symbiotic performance [61]. Accordingly, genes encoding the putative KdpABC pump were also present within *Azospirillum* genomes forming a putative operon with *kdpD* and *kdpE* (Table 6). The Kdp system has been shown to be crucial in the regulation of many host-pathogen interaction mechanisms, promoting bacterial virulence and resistance against various stresses [62]. Therefore, the *Azospirillum* KdpD/KdpE system may confer an adaptive advantage to *Azospirillum* in the rhizosphere.

**Table 6:** *Azospirillum* TCS orthologues with inferred biological functions

Gene^a^	Label^b^	BBH (% amino acid identity)^c^	Putative functions	Relevant feature	References
*chvG chvI*	AZOLI_2965 AZOLI_2966	*Rhodospirillum centenum* (60 %) *Rhodospirillum centenum* (84 %)	Virulence and TVISS regulation in *A. tumefaciens* Biofilm and motility in *S. meliloti*	Conserved in members of the α-proteobacteria	[68] [66]
*phoR phoB*	AZOLI_3010 AZOLI_3109	*Rhodospirillum centenum* (54 %) *Rhodopseudomonas palustris* (65 %)	Virulence and survival in *Vibrio cholerae* Biofilm formation in *P. fluorescens*	*phoB* located upstream a putative *pst* ABC transporter	[69] [64]
*dctB dctD*	AZOLI_p10629 AZOLI_p10628	*Arhodomonas aquaeolei* (60 %) *Pseudomonas fuscovaginae* (43 %)	Root colonization and beneficial properties in *P. chlororaphis* C4-dicarboxylate transport and nitrogen fixation in rhizobia	Located upstream of a putative *dctA* transporter	[70] [71]
*ntrB ntrC*	AZOLI_1342 AZOLI_1343	*Rhodospirillum centenum* (73 %) *Rhodospirillum centenum* (83 %)	Regulation of nitrogen metabolism, nitrate utilization and ammonium transport	Clustered with *ntrXY*	[59]
*ntrX ntrY*	AZOLI_1344 AZOLI_1345	*Rhodospirillum centenum* (62 %) *Rhodospirillum centenum* (83 %)	Regulation of nitrogen metabolism, nitrate utilization and ammonium transport	Clustered with *ntrBC*	[60]
*kdpD kdpE*	AZOLI_p20517 AZOLI_p20518	*Pseudaminobacter salicylatoxidans* (76 %) *Rhodospirillum bacterium* URHD0088 (64 %)	Potassium transport and virulence in *S. aureus* Osmoadaptation and symbiosis in *S. meliloti*	Located next to *kdpABC* encoding a potassium pump	[72] [61]
*lytS lytR*	AZOLI_p50421 AZOLI_p50422	*Rhodospirillum rubrum* (74 %) *Rhodospirillum rubrum* (73 %)	Carbon control network in *E. coli* Virulence in *S. aureus*	Located next to genes encoding a putative MDR efflux pump	[73] [67]
*flcA*	AZOLI_1403	*Rhodospirillum centenum* (65 %)	Flocculation and root colonization in *A. brasilense*	Orphan RR	[74]
*bphP1*	AZOLI_p20424	*Stenotrophomonas maltophilia* (53 %)	Light-stress tolerance and motility in *A. brasilense*	Organized in a triad of TCS (HyHK-RR-Bph1)	[48]

^a^For each pair, the first gene encodes the HK whereas the second gene encodes the RR

^b^Labels refer to genes of *A. lipoferum* 4B. Labels of the orthologues of other *Azospirillum* strains can be found in Additional file 3: Table S2**;** Additional file 4: Table S3; Additional file 5: Table S4 and Additional file 6: Table S5

^c^ *A. lipoferum* 4B amino acid sequences were used as queries

TCS sharing similarity with the PhoB/PhoR signal transduction system were also retrieved from all *Azospirillum* genomes, adjacent to the *pstSCAB-phoU* operon encoding an ABC transporter important for the high-affinity capture of periplasmic inorganic phosphate (Table 6) [63]. Under low inorganic phosphate concentration, PhoB/PhoR activates the *pstSCAB-phoU* operon [63]. Moreover, a link between the regulation of phosphate metabolism and biofilm formation has been highlighted in some rhizobacteria, as expression of the Pho regulon was shown to affect biofilm formation in *P. fluorescens* [64].

Homologues of the *chvG/chvI* genes are also present in the four *Azospirillum* genomes (Table 6). This TCS is highly conserved among free-living alphaproteobacteria where it controls many processes involved in successful host-bacteria interaction. Indeed, ChvGI regulates the expression of virulence gene expression in response to acidic conditions in the plant pathogen *Agrobacterium tumefaciens* [65] whereas in *S. meliloti* ExoS, an orthologue of ChvG, positively regulates succinoglycan synthesis (thereby promoting biofilm formation) and prevents flagellum biosynthesis [66]. However, to our knowledge whether this TCS in *Azospirillum* plays a role in the plant-bacteria interaction has never been elucidated.

Interestingly, one *Azospirillum* TCS belongs to the family of LytS/LytR TCS, shown to be involved in the regulation of bacterial virulence genes (Table 6) [67]. Based on sequence analyses, the LytS-like HK is predicted to be a membrane-bound sensor, and the LytR-like RR is predicted to contain a CheY-like REC domain and a DNA-binding domain (PF04397), as expected. The analysis of the genetic context of the LytS/LytR-like genes in *Azospirillum* showed a genetic link with genes encoding a putative multidrug resistance efflux pump. To our knowledge, whether the LytS/LytR-like in *Azospirillum* is functionally coupled to the regulation of the adjacent efflux pump has never been investigated.

However, there are differences in TCS proteins among the four *Azospirillum* strains that may contribute to niche-specific adaptation. The number of strain-specific TCS genes varies greatly among the four genomes (Fig. 4; Additional 3: Table S2; Additional file 4: Table S3; Additional file 5: Table S4; and Additional file 6: Table S5). For the vast majority of these unique TCS genes, making functional correlations seems hazardous but several unique TCS genes of *A. lipoferum* 4B caught our attention. In this strain, the five first genes of the TCS hexad, spanning from AZOLI_0973 to AZOLI_0977, had no putative homologue in the three other strains. Similarity searches of these genes revealed significant matches with non-contiguous TCS genes of *M. magneticum*, except for AZOLI_0976 which encodes an atypical RR (REC-PAS-GGDEF) showing a significant identity (41 %) over the PAS and GGDEF domains with a protein from psychrophile *Colwellia psychrerythraea* belonging to the gammaproteobacteria lineage (Additional file 3: Table S2). Two explanations can be proposed to explain these observations. First, the presence of these five genes in *A. lipoferum* 4B and *M. magneticum* may result from HGT between these two strains, or alternatively, they may have been present in the common ancestor shared by these two strains, and secondarily lost in the three others *Azospirillum* strains and in *Rhodospirillum centenum* SW in agreement with the phylogeny of species (Additional file 2: Figure S1). As for the proteins encoded by AZOLI_p10944 and AZOLI_p10945, they exhibit homology to the MoxYX system of *Starkeya novella*, a facultative chemolithoautotrophic and methylotrophic alphaproteobacterium (Additional file 3: Table S2). Synteny analysis between these two genomic regions revealed strong conservation of the *moxXY* genes forming a putative operon with *moxFJGIR*, thus suggesting that MoxYX might control *A. lipoferum* 4B methanol metabolism. Interestingly, *A. lipoferum* 4B was shown to grow slightly with methanol as sole carbon source (Our unpublished data). Therefore, tight regulation of this metabolic versatility may be relevant in specific environments such as rice paddy fields, from which *A. lipoferum* 4B was isolated.

## Conclusions

In summary, here we provide evidence of the incredible complexity of *Azospirillum* TCS regulatory networks. The existence of large numbers of TCS and especially HyHKs reinforces the idea that these genes can support traits needed for thriving in heterogeneous, fluctuating and highly competitive environments, such as the rhizosphere. Our phylogenetic analyses indicate that the expansion of the HyHK family in *Azospirillum* has occurred mainly through HGT, even if gene duplication and domain shuffling also played an important role. We further gained insights into the core of orthologues TCS proteins and differences between TCS repertoire of the four strains that are likely to facilitate *Azospirillum* adaptation to different host plants. Future studies focused on the functional analysis of these TCS might shed light on the complex events occurring during *Azospirillum* soil survival and plant growth-promotion in different environmental settings. Ultimately, focusing on mechanisms by which beneficial plant-associated bacteria persist and compete in soil would be useful for improving plant growth-promotion strategies.

## Additional files

Additional file 1: Table S1. Complete list of genomes surveyed during this study. (XLSX 153 kb) Additional file 2: Figure S1. Distance-based phylogenetic tree showing the relationships among the different organisms being considered in this study. The phylogeny was derived from concatenated alignments of the ribosomal proteins L3, L5, L11, L13, L14, S3, S7, S9, S11, and S17. Numbers at branch correspond to bootstrap values (1,000 replicates of the original alignment). Branch lengths are proportional to the number of substitutions per site (see the scale bar). Blue boxes highlight alpha-proteobacterial sequences that are closely related to that of *A. lipoferum* (represented in blue). **Figure S2.** Phylogenies of the transmitter and REC domain of AZOLI_p30008. Maximum Likelihood trees of the transmitter (A) and REC (B) domains of the AZOLI_p30008 (153 and 83 amino acid positions, respectively). Numbers at branch correspond to SH-like supports calculated with PHYML (for clarity values lesser than 0.90 are not shown). The scale bars indicate the average number of substitution per site. Colours correspond to taxonomic groups: Orange: *A. lipoferum* 4B, light blue: *Azospirillum* sp. B510, light green: *A. brasilense* CBG497, dark green: *A. brasilense* Sp245, dark blue: other alphaproteobacteria, light brown: *Delta*/*Epsilonproteobacteria*, pink: *Betaproteobacteria*, dark brown: *Gammaproteobacteria*, black: other bacteria. The proximity of both domains with sequences from *Methylobacteriuym* suggests that a horizontal gene transfer event occurred between these two lineages. **Figure S3.** Phylogenies of the transmitter and REC domains of AZOLI_p10058. Maximum Likelihood trees of the transmitter (A) and REC (B) domains of the AZOLI_p10058 (157 and 90 amino acid positions, respectively). Numbers at branch correspond to SH-like supports calculated with PHYML (for clarity values lesser than 0.90 are not shown). The scale bars indicate the average number of substitution per site. Colours correspond to taxonomic groups: Orange: *A. lipoferum* 4B, light blue: *Azospirillum* sp. B510, light green: *A. brasilense* CBG497, dark green: *A. brasilense* Sp245, dark blue: other alphaproteobacteria, light brown: *Delta*/*Epsilonproteobacteria*, pink: *Betaproteobacteria*, dark brown: *Gammaproteobacteria*, black: other bacteria. A duplication event leading to *Azospirillum* sp. B510 YP_003452479 and *A. lipoferum* 4B YP_004975186 on the one hand, and to *Azospirillum* sp. B510 YP_003450084 and *A. lipoferum* 4B YP_004973569 on the other hand is visible on both trees. (PPT 820 kb) Additional file 3: Table S2. Domain architecture, genetic organization and subcellular localization of *A. lipoferum* 4B TCS components. (XLSX 40 kb) Additional file 4: Table S3. Domain architecture, genetic organization and subcellular localization of *Azospirillum* sp. B510 TCS components. (XLSX 72 kb) Additional file 5: Table S4. Domain architecture, genetic organization and subcellular localization of *A. brasilense* Sp245 TCS components. (XLSX 33 kb) Additional file 6: Table S5. Domain architecture, genetic organization and subcellular localization of *A. brasilense* CBG497 TCS components. (XLSX 28 kb)

## Abbreviations

TCS: Two-component systems
HK: Histidine kinase
HyHK: Hybrid histidine kinase
LSE: Lineage specific expansion
REC: Receiver domain
RR: Response regulator
