# Supplementary material for: Genome-wide survey of two-component signal transduction systems in the plant growth-promoting bacterium Azospirillum
Source: BMC Genomics. 2015 Oct 22;16:833. doi: 10.1186/s12864-015-1962-x (PMC4618731; doi:10.1186/s12864-015-1962-x)
Supplement: Additional file 2: Figure S1. — Distance-based phylogenetic tree showing the relationships among the different organisms being considered in this study. The phylogeny was derived from concatenated alignments of the ribosomal proteins L3, L5, L11, L13, L14, S3, S7, S9, S11, and S17. Numbers at branch correspond to bootstrap values (1,000 replicates of the original alignment). Branch lengths are proportional to the number of substitutions per site (see the scale bar). Blue boxes highlight alpha-proteobacterial sequences that are closely related to that of A. lipoferum (represented in blue). Figure S2. Phylogenies of the transmitter and REC domain of AZOLI_p30008. Maximum Likelihood trees of the transmitter (A) and REC (B) domains of the AZOLI_p30008 (153 and 83 amino acid positions, respectively). Numbers at branch correspond to SH-like supports calculated with PHYML (for clarity values lesser than 0.90 are not shown). The scale bars indicate the average number of substitution per site. Colours correspond to taxonomic groups: Orange: A. lipoferum 4B, light blue: Azospirillum sp. B510, light green: A. brasilense CBG497, dark green: A. brasilense Sp245, dark blue: other alphaproteobacteria, light brown: Delta/Epsilonproteobacteria, pink: Betaproteobacteria, dark brown: Gammaproteobacteria, black: other bacteria. The proximity of both domains with sequences from Methylobacteriuym suggests that a horizontal gene transfer event occurred between these two lineages. Figure S3. Phylogenies of the transmitter and REC domains of AZOLI_p10058. Maximum Likelihood trees of the transmitter (A) and REC (B) domains of the AZOLI_p10058 (157 and 90 amino acid positions, respectively). Numbers at branch correspond to SH-like supports calculated with PHYML (for clarity values lesser than 0.90 are not shown). The scale bars indicate the average number of substitution per site. Colours correspond to taxonomic groups: Orange: A. lipoferum 4B, light blue: Azospirillum sp. B510, light green: A. brasilense CBG497, dark green [file 12864_2015_1962_MOESM2_ESM.ppt]

## Slide 1
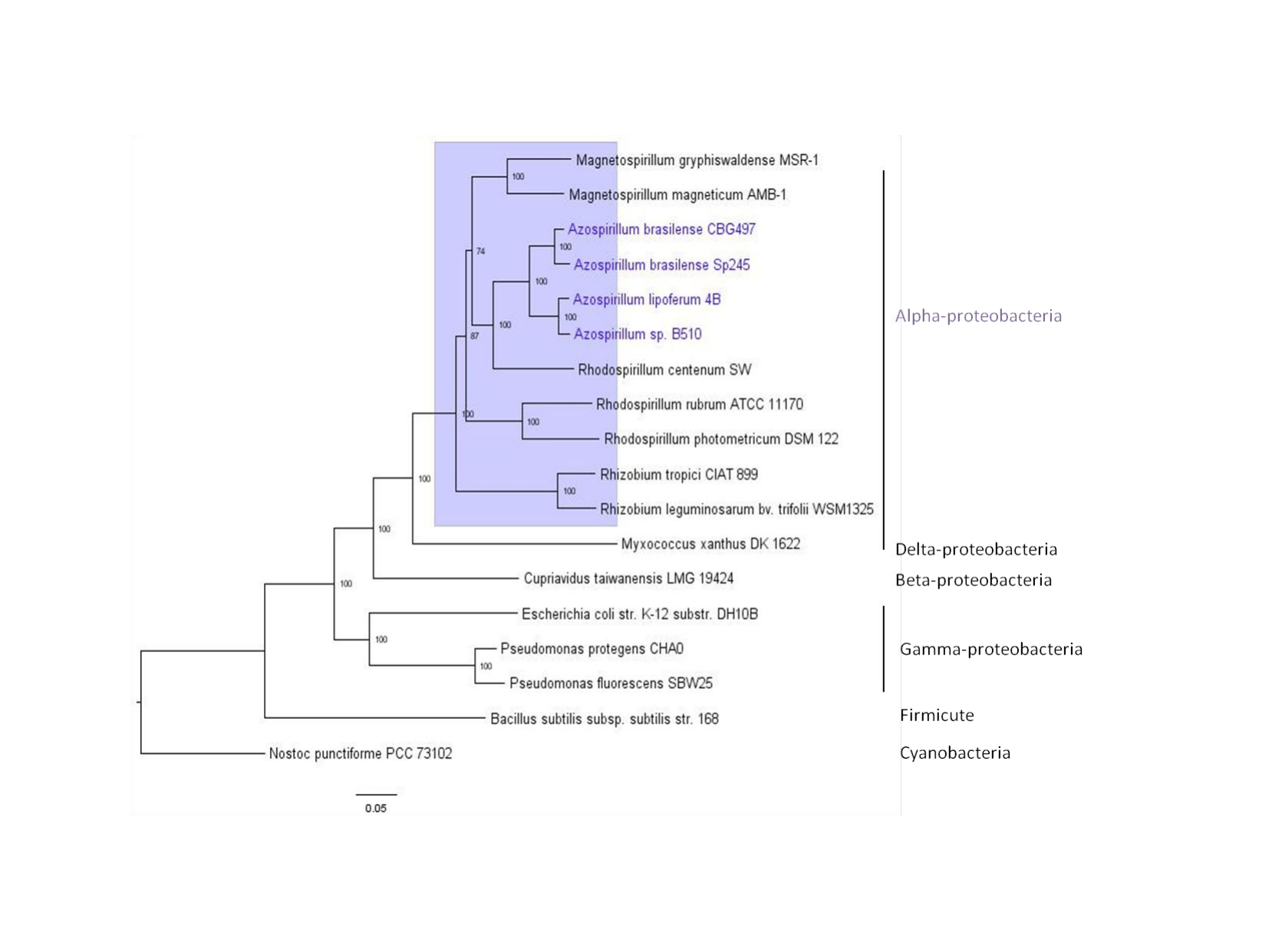

## Slide 2
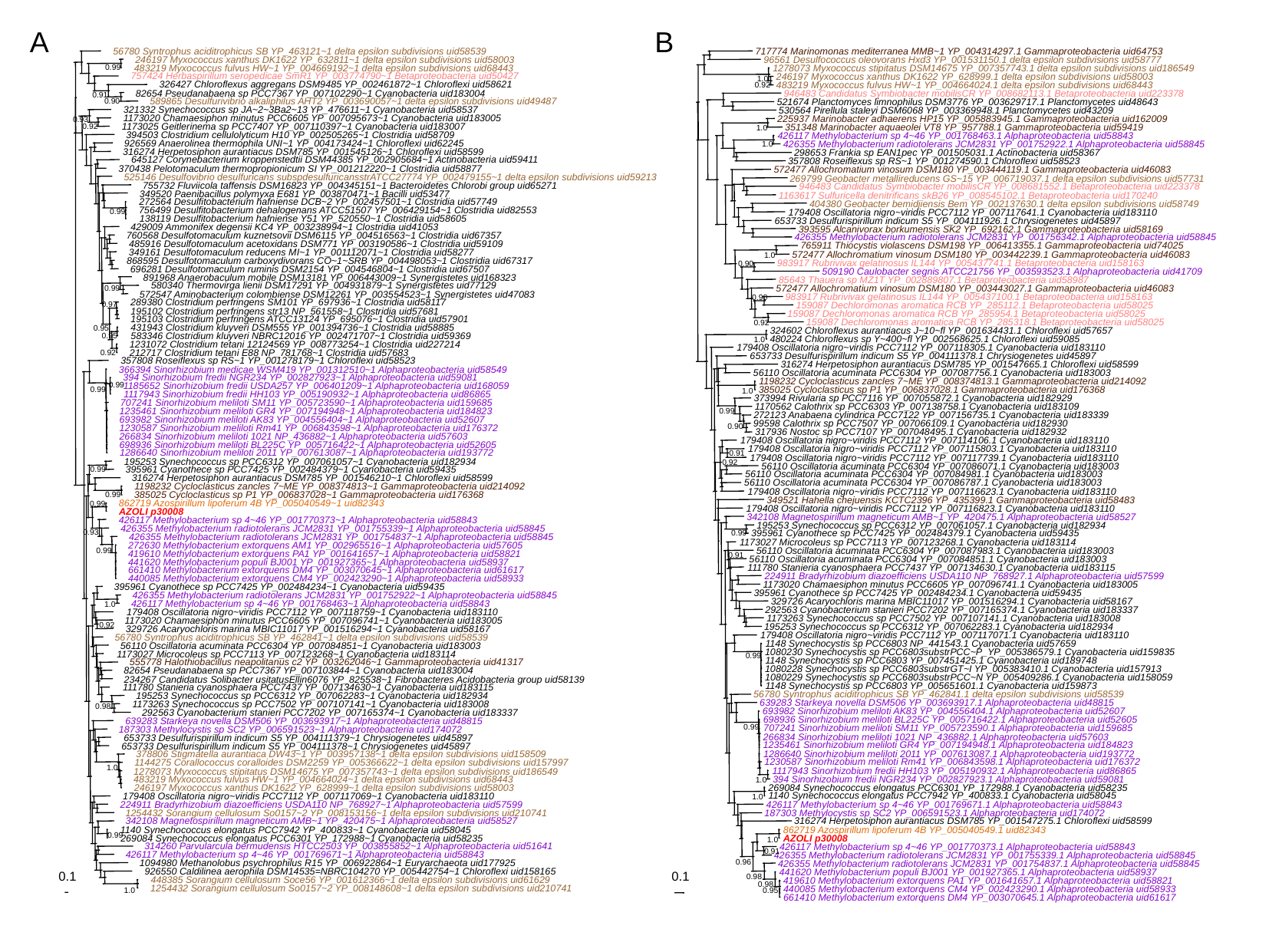

A
B
56780 Syntrophus aciditrophicus SB YP_463121~1 delta epsilon subdivisions uid58539
717774 Marinomonas mediterranea MMB~1 YP_004314297.1 Gammaproteobacteria uid64753
246197 Myxococcus xanthus DK1622 YP_632811~1 delta epsilon subdivisions uid58003
96561 Desulfococcus oleovorans Hxd3 YP_001531150.1 delta epsilon subdivisions uid58777
483219 Myxococcus fulvus HW~1 YP_004669192~1 delta epsilon subdivisions uid68443
1278073 Myxococcus stipitatus DSM14675 YP_007357743.1 delta epsilon subdivisions uid186549
0.99
757424 Herbaspirillum seropedicae SmR1 YP_003774790~1 Betaproteobacteria uid50427
246197 Myxococcus xanthus DK1622 YP_628999.1 delta epsilon subdivisions uid58003
1.0
326427 Chloroflexus aggregans DSM9485 YP_002461872~1 Chloroflexi uid58621
483219 Myxococcus fulvus HW~1 YP_004664024.1 delta epsilon subdivisions uid68443
0.92
82654 Pseudanabaena sp PCC7367 YP_007102290~1 Cyanobacteria uid183004
946483 Candidatus Symbiobacter mobilisCR YP_008682113.1 Betaproteobacteria uid223378
0.91
589865 Desulfurivibrio alkaliphilus AHT2 YP_003690057~1 delta epsilon subdivisions uid49487
0.90
521674 Planctomyces limnophilus DSM3776 YP_003629717.1 Planctomycetes uid48643
321332 Synechococcus sp JA~2~3Ba2~13 YP_476611~1 Cyanobacteria uid58537
530564 Pirellula staleyi DSM6068 YP_003369948.1 Planctomycetes uid43209
1173020 Chamaesiphon minutus PCC6605 YP_007095673~1 Cyanobacteria uid183005
225937 Marinobacter adhaerens HP15 YP_005883945.1 Gammaproteobacteria uid162009
0.93
1173025 Geitlerinema sp PCC7407 YP_007110397~1 Cyanobacteria uid183007
0.92
351348 Marinobacter aquaeolei VT8 YP_957788.1 Gammaproteobacteria uid59419
1.0
394503 Clostridium cellulolyticum H10 YP_002505265~1 Clostridia uid58709
426117 Methylobacterium sp 4~46 YP_001768463.1 Alphaproteobacteria uid58843
926569 Anaerolinea thermophila UNI~1 YP_004173424~1 Chloroflexi uid62245
426355 Methylobacterium radiotolerans JCM2831 YP_001752922.1 Alphaproteobacteria uid58845
1.0
316274 Herpetosiphon aurantiacus DSM785 YP_001545126~1 Chloroflexi uid58599
298653 Frankia sp EAN1pec YP_001505031.1 Actinobacteria uid58367
645127 Corynebacterium kroppenstedtii DSM44385 YP_002905684~1 Actinobacteria uid59411
357808 Roseiflexus sp RS~1 YP_001274590.1 Chloroflexi uid58523
370438 Pelotomaculum thermopropionicum SI YP_001212220~1 Clostridia uid58877
572477 Allochromatium vinosum DSM180 YP_003444119.1 Gammaproteobacteria uid46083
525146 Desulfovibrio desulfuricans subspdesulfuricansstrATCC27774 YP_002479155~1 delta epsilon subdivisions uid59213
269799 Geobacter metallireducens GS~15 YP_006719037.1 delta epsilon subdivisions uid57731
755732 Fluviicola taffensis DSM16823 YP_004345151~1 Bacteroidetes Chlorobi group uid65271
946483 Candidatus Symbiobacter mobilisCR YP_008681552.1 Betaproteobacteria uid223378
349520 Paenibacillus polymyxa E681 YP_003870471~1 Bacilli uid53477
1163617 Sulfuricella denitrificans skB26 YP_008545102.1 Betaproteobacteria uid170240
272564 Desulfitobacterium hafniense DCB~2 YP_002457501~1 Clostridia uid57749
404380 Geobacter bemidjiensis Bem YP_002137630.1 delta epsilon subdivisions uid58749
756499 Desulfitobacterium dehalogenans ATCC51507 YP_006429154~1 Clostridia uid82553
179408 Oscillatoria nigro~viridis PCC7112 YP_007117641.1 Cyanobacteria uid183110
0.99
138119 Desulfitobacterium hafniense Y51 YP_520550~1 Clostridia uid58605
653733 Desulfurispirillum indicum S5 YP_004111926.1 Chrysiogenetes uid45897
429009 Ammonifex degensii KC4 YP_003238994~1 Clostridia uid41053
393595 Alcanivorax borkumensis SK2 YP_692162.1 Gammaproteobacteria uid58169
760568 Desulfotomaculum kuznetsovii DSM6115 YP_004516563~1 Clostridia uid67357
426355 Methylobacterium radiotolerans JCM2831 YP_001756342.1 Alphaproteobacteria uid58845
485916 Desulfotomaculum acetoxidans DSM771 YP_003190586~1 Clostridia uid59109
765911 Thiocystis violascens DSM198 YP_006413355.1 Gammaproteobacteria uid74025
349161 Desulfotomaculum reducens MI~1 YP_001112071~1 Clostridia uid58277
572477 Allochromatium vinosum DSM180 YP_003442239.1 Gammaproteobacteria uid46083
1.0
868595 Desulfotomaculum carboxydivorans CO~1~SRB YP_004498053~1 Clostridia uid67317
983917 Rubrivivax gelatinosus IL144 YP_005437741.1 Betaproteobacteria uid158163
0.90
696281 Desulfotomaculum ruminis DSM2154 YP_004546804~1 Clostridia uid67507
509190 Caulobacter segnis ATCC21756 YP_003593523.1 Alphaproteobacteria uid41709
891968 Anaerobaculum mobile DSM13181 YP_006443009~1 Synergistetes uid168323
85643 Thauera sp MZ1T YP_002889807.1 Betaproteobacteria uid58987
580340 Thermovirga lienii DSM17291 YP_004931879~1 Synergistetes uid77129
572477 Allochromatium vinosum DSM180 YP_003443027.1 Gammaproteobacteria uid46083
0.99
572547 Aminobacterium colombiense DSM12261 YP_003554523~1 Synergistetes uid47083
983917 Rubrivivax gelatinosus IL144 YP_005437100.1 Betaproteobacteria uid158163
0.90
289380 Clostridium perfringens SM101 YP_697936~1 Clostridia uid58117
159087 Dechloromonas aromatica RCB YP_285112.1 Betaproteobacteria uid58025
0.97
195102 Clostridium perfringens str13 NP_561558~1 Clostridia uid57681
159087 Dechloromonas aromatica RCB YP_285954.1 Betaproteobacteria uid58025
195103 Clostridium perfringens ATCC13124 YP_695076~1 Clostridia uid57901
159087 Dechloromonas aromatica RCB YP_285318.1 Betaproteobacteria uid58025
0.92
431943 Clostridium kluyveri DSM555 YP_001394736~1 Clostridia uid58885
0.95
324602 Chloroflexus aurantiacus J~10~fl YP_001634431.1 Chloroflexi uid57657
583346 Clostridium kluyveri NBRC12016 YP_002471707~1 Clostridia uid59369
0.95
480224 Chloroflexus sp Y~400~fl YP_002568625.1 Chloroflexi uid59085
1.0
1231072 Clostridium tetani 12124569 YP_008773254~1 Clostridia uid227214
179408 Oscillatoria nigro~viridis PCC7112 YP_007118305.1 Cyanobacteria uid183110
212717 Clostridium tetani E88 NP_781768~1 Clostridia uid57683
0.92
653733 Desulfurispirillum indicum S5 YP_004111378.1 Chrysiogenetes uid45897
357808 Roseiflexus sp RS~1 YP_001278179~1 Chloroflexi uid58523
316274 Herpetosiphon aurantiacus DSM785 YP_001547665.1 Chloroflexi uid58599
366394 Sinorhizobium medicae WSM419 YP_001312510~1 Alphaproteobacteria uid58549
56110 Oscillatoria acuminata PCC6304 YP_007087756.1 Cyanobacteria uid183003
394 Sinorhizobium fredii NGR234 YP_002827923~1 Alphaproteobacteria uid59081
1198232 Cycloclasticus zancles 7~ME YP_008374813.1 Gammaproteobacteria uid214092
0.99
1185652 Sinorhizobium fredii USDA257 YP_006401209~1 Alphaproteobacteria uid168059
385025 Cycloclasticus sp P1 YP_006837028.1 Gammaproteobacteria uid176368
0.99
1.0
1117943 Sinorhizobium fredii HH103 YP_005190932~1 Alphaproteobacteria uid86865
373994 Rivularia sp PCC7116 YP_007055872.1 Cyanobacteria uid182929
707241 Sinorhizobium meliloti SM11 YP_005723590~1 Alphaproteobacteria uid159685
1170562 Calothrix sp PCC6303 YP_007138758.1 Cyanobacteria uid183109
1235461 Sinorhizobium meliloti GR4 YP_007194948~1 Alphaproteobacteria uid184823
0.99
272123 Anabaena cylindrica PCC7122 YP_007156735.1 Cyanobacteria uid183339
693982 Sinorhizobium meliloti AK83 YP_004556404~1 Alphaproteobacteria uid52607
99598 Calothrix sp PCC7507 YP_007066109.1 Cyanobacteria uid182930
0.90
1230587 Sinorhizobium meliloti Rm41 YP_006843598~1 Alphaproteobacteria uid176372
317936 Nostoc sp PCC7107 YP_007048495.1 Cyanobacteria uid182932
266834 Sinorhizobium meliloti 1021 NP_436882~1 Alphaproteobacteria uid57603
179408 Oscillatoria nigro~viridis PCC7112 YP_007114106.1 Cyanobacteria uid183110
698936 Sinorhizobium meliloti BL225C YP_005716422~1 Alphaproteobacteria uid52605
179408 Oscillatoria nigro~viridis PCC7112 YP_007115803.1 Cyanobacteria uid183110
1286640 Sinorhizobium meliloti 2011 YP_007613087~1 Alphaproteobacteria uid193772
0.91
179408 Oscillatoria nigro~viridis PCC7112 YP_007117739.1 Cyanobacteria uid183110
195253 Synechococcus sp PCC6312 YP_007061057~1 Cyanobacteria uid182934
0.92
56110 Oscillatoria acuminata PCC6304 YP_007086071.1 Cyanobacteria uid183003
395961 Cyanothece sp PCC7425 YP_002484379~1 Cyanobacteria uid59435
0.99
56110 Oscillatoria acuminata PCC6304 YP_007084981.1 Cyanobacteria uid183003
316274 Herpetosiphon aurantiacus DSM785 YP_001546210~1 Chloroflexi uid58599
56110 Oscillatoria acuminata PCC6304 YP_007086787.1 Cyanobacteria uid183003
1198232 Cycloclasticus zancles 7~ME YP_008374813~1 Gammaproteobacteria uid214092
179408 Oscillatoria nigro~viridis PCC7112 YP_007116623.1 Cyanobacteria uid183110
385025 Cycloclasticus sp P1 YP_006837028~1 Gammaproteobacteria uid176368
0.99
349521 Hahella chejuensis KCTC2396 YP_435399.1 Gammaproteobacteria uid58483
862719 Azospirillum lipoferum 4B YP_005040549~1 uid82343
0.99
179408 Oscillatoria nigro~viridis PCC7112 YP_007116823.1 Cyanobacteria uid183110
AZOLI p30008
342108 Magnetospirillum magneticum AMB~1 YP_420475.1 Alphaproteobacteria uid58527
426117 Methylobacterium sp 4~46 YP_001770373~1 Alphaproteobacteria uid58843
195253 Synechococcus sp PCC6312 YP_007061057.1 Cyanobacteria uid182934
426355 Methylobacterium radiotolerans JCM2831 YP_001755339~1 Alphaproteobacteria uid58845
0.93
395961 Cyanothece sp PCC7425 YP_002484379.1 Cyanobacteria uid59435
0.99
426355 Methylobacterium radiotolerans JCM2831 YP_001754837~1 Alphaproteobacteria uid58845
1173027 Microcoleus sp PCC7113 YP_007123268.1 Cyanobacteria uid183114
272630 Methylobacterium extorquens AM1 YP_002965516~1 Alphaproteobacteria uid57605
56110 Oscillatoria acuminata PCC6304 YP_007087983.1 Cyanobacteria uid183003
0.99
419610 Methylobacterium extorquens PA1 YP_001641657~1 Alphaproteobacteria uid58821
0.91
56110 Oscillatoria acuminata PCC6304 YP_007084851.1 Cyanobacteria uid183003
441620 Methylobacterium populi BJ001 YP_001927365~1 Alphaproteobacteria uid58937
111780 Stanieria cyanosphaera PCC7437 YP_007134630.1 Cyanobacteria uid183115
661410 Methylobacterium extorquens DM4 YP_003070645~1 Alphaproteobacteria uid61617
224911 Bradyrhizobium diazoefficiens USDA110 NP_768927.1 Alphaproteobacteria uid57599
440085 Methylobacterium extorquens CM4 YP_002423290~1 Alphaproteobacteria uid58933
1173020 Chamaesiphon minutus PCC6605 YP_007096741.1 Cyanobacteria uid183005
395961 Cyanothece sp PCC7425 YP_002484234~1 Cyanobacteria uid59435
395961 Cyanothece sp PCC7425 YP_002484234.1 Cyanobacteria uid59435
426355 Methylobacterium radiotolerans JCM2831 YP_001752922~1 Alphaproteobacteria uid58845
329726 Acaryochloris marina MBIC11017 YP_001516294.1 Cyanobacteria uid58167
426117 Methylobacterium sp 4~46 YP_001768463~1 Alphaproteobacteria uid58843
1.0
292563 Cyanobacterium stanieri PCC7202 YP_007165374.1 Cyanobacteria uid183337
179408 Oscillatoria nigro~viridis PCC7112 YP_007118759~1 Cyanobacteria uid183110
1173263 Synechococcus sp PCC7502 YP_007107141.1 Cyanobacteria uid183008
1173020 Chamaesiphon minutus PCC6605 YP_007096741~1 Cyanobacteria uid183005
0.92
195253 Synechococcus sp PCC6312 YP_007062283.1 Cyanobacteria uid182934
329726 Acaryochloris marina MBIC11017 YP_001516294~1 Cyanobacteria uid58167
179408 Oscillatoria nigro~viridis PCC7112 YP_007117071.1 Cyanobacteria uid183110
56780 Syntrophus aciditrophicus SB YP_462841~1 delta epsilon subdivisions uid58539
1148 Synechocystis sp PCC6803 NP_441543.1 Cyanobacteria uid57659
56110 Oscillatoria acuminata PCC6304 YP_007084851~1 Cyanobacteria uid183003
1080230 Synechocystis sp PCC6803substrPCC~P_YP_005386579.1 Cyanobacteria uid159835
1173027 Microcoleus sp PCC7113 YP_007123268~1 Cyanobacteria uid183114
0.99
1148 Synechocystis sp PCC6803 YP_007451425.1 Cyanobacteria uid189748
555778 Halothiobacillus neapolitanus c2 YP_003262046~1 Gammaproteobacteria uid41317
1080228 Synechocystis sp PCC6803substrGT~I YP_005383410.1 Cyanobacteria uid157913
82654 Pseudanabaena sp PCC7367 YP_007103844~1 Cyanobacteria uid183004
1080229 Synechocystis sp PCC6803substrPCC~N YP_005409286.1 Cyanobacteria uid158059
234267 Candidatus Solibacter usitatusEllin6076 YP_825538~1 Fibrobacteres Acidobacteria group uid58139
1148 Synechocystis sp PCC6803 YP_005651601.1 Cyanobacteria uid159873
111780 Stanieria cyanosphaera PCC7437 YP_007134630~1 Cyanobacteria uid183115
56780 Syntrophus aciditrophicus SB YP_462841.1 delta epsilon subdivisions uid58539
195253 Synechococcus sp PCC6312 YP_007062283~1 Cyanobacteria uid182934
639283 Starkeya novella DSM506 YP_003693917.1 Alphaproteobacteria uid48815
1173263 Synechococcus sp PCC7502 YP_007107141~1 Cyanobacteria uid183008
0.98
693982 Sinorhizobium meliloti AK83 YP_004556404.1 Alphaproteobacteria uid52607
292563 Cyanobacterium stanieri PCC7202 YP_007165374~1 Cyanobacteria uid183337
698936 Sinorhizobium meliloti BL225C YP_005716422.1 Alphaproteobacteria uid52605
639283 Starkeya novella DSM506 YP_003693917~1 Alphaproteobacteria uid48815
0.99
707241 Sinorhizobium meliloti SM11 YP_005723590.1 Alphaproteobacteria uid159685
187303 Methylocystis sp SC2 YP_006591523~1 Alphaproteobacteria uid174072
266834 Sinorhizobium meliloti 1021 NP_436882.1 Alphaproteobacteria uid57603
653733 Desulfurispirillum indicum S5 YP_004111379~1 Chrysiogenetes uid45897
1235461 Sinorhizobium meliloti GR4 YP_007194948.1 Alphaproteobacteria uid184823
653733 Desulfurispirillum indicum S5 YP_004111378~1 Chrysiogenetes uid45897
1286640 Sinorhizobium meliloti 2011 YP_007613087.1 Alphaproteobacteria uid193772
378806 Stigmatella aurantiaca DW43~1 YP_003957138~1 delta epsilon subdivisions uid158509
1230587 Sinorhizobium meliloti Rm41 YP_006843598.1 Alphaproteobacteria uid176372
1144275 Corallococcus coralloides DSM2259 YP_005366622~1 delta epsilon subdivisions uid157997
1.0
1117943 Sinorhizobium fredii HH103 YP_005190932.1 Alphaproteobacteria uid86865
1278073 Myxococcus stipitatus DSM14675 YP_007357743~1 delta epsilon subdivisions uid186549
394 Sinorhizobium fredii NGR234 YP_002827923.1 Alphaproteobacteria uid59081
483219 Myxococcus fulvus HW~1 YP_004664024~1 delta epsilon subdivisions uid68443
1.0
269084 Synechococcus elongatus PCC6301 YP_172988.1 Cyanobacteria uid58235
246197 Myxococcus xanthus DK1622 YP_628999~1 delta epsilon subdivisions uid58003
1140 Synechococcus elongatus PCC7942 YP_400833.1 Cyanobacteria uid58045
179408 Oscillatoria nigro~viridis PCC7112 YP_007117069~1 Cyanobacteria uid183110
1.0
426117 Methylobacterium sp 4~46 YP_001769671.1 Alphaproteobacteria uid58843
224911 Bradyrhizobium diazoefficiens USDA110 NP_768927~1 Alphaproteobacteria uid57599
1254432 Sorangium cellulosum So0157~2 YP_008153156~1 delta epsilon subdivisions uid210741
187303 Methylocystis sp SC2 YP_006591523.1 Alphaproteobacteria uid174072
342108 Magnetospirillum magneticum AMB~1 YP_420475~1 Alphaproteobacteria uid58527
316274 Herpetosiphon aurantiacus DSM785 YP_001547275.1 Chloroflexi uid58599
1140 Synechococcus elongatus PCC7942 YP_400833~1 Cyanobacteria uid58045
862719 Azospirillum lipoferum 4B YP_005040549.1 uid82343
0.99
269084 Synechococcus elongatus PCC6301 YP_172988~1 Cyanobacteria uid58235
AZOLI p30008
1.0
314260 Parvularcula bermudensis HTCC2503 YP_003855852~1 Alphaproteobacteria uid51641
426117 Methylobacterium sp 4~46 YP_001770373.1 Alphaproteobacteria uid58843
0.91
426117 Methylobacterium sp 4~46 YP_001769671~1 Alphaproteobacteria uid58843
426355 Methylobacterium radiotolerans JCM2831 YP_001755339.1 Alphaproteobacteria uid58845
0.96
1094980 Methanolobus psychrophilus R15 YP_006922864~1 Euryarchaeota uid177925
426355 Methylobacterium radiotolerans JCM2831 YP_001754837.1 Alphaproteobacteria uid58845
926550 Caldilinea aerophila DSM14535=NBRC104270 YP_005442754~1 Chloroflexi uid158165
441620 Methylobacterium populi BJ001 YP_001927365.1 Alphaproteobacteria uid58937
0.1
0.1
0.98
448385 Sorangium cellulosum Soce56 YP_001612366~1 delta epsilon subdivisions uid61629
419610 Methylobacterium extorquens PA1 YP_001641657.1 Alphaproteobacteria uid58821
0.98
1254432 Sorangium cellulosum So0157~2 YP_008148608~1 delta epsilon subdivisions uid210741
440085 Methylobacterium extorquens CM4 YP_002423290.1 Alphaproteobacteria uid58933
1.0
0.95
661410 Methylobacterium extorquens DM4 YP_003070645.1 Alphaproteobacteria uid61617

## Slide 3
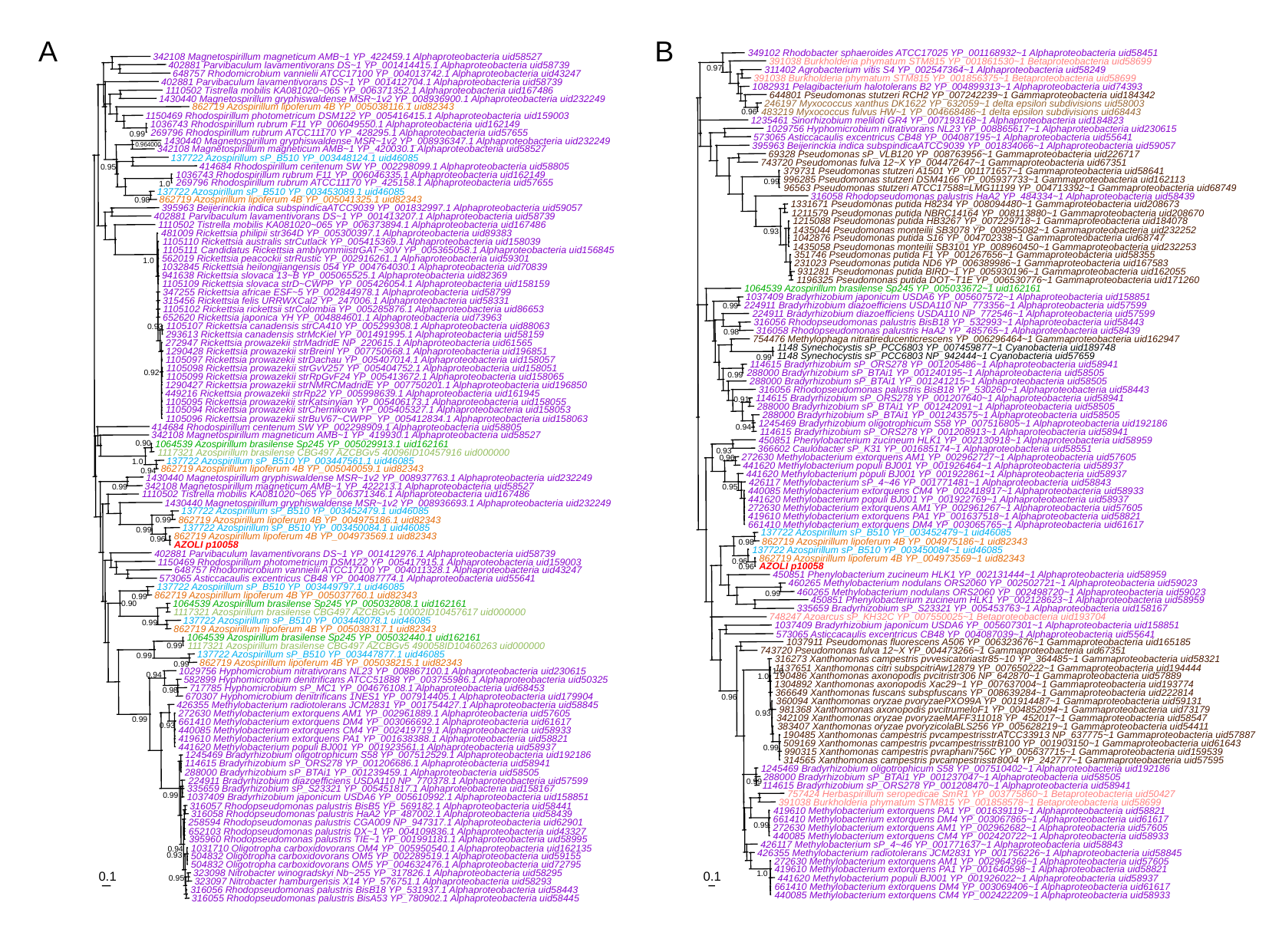

A
B
349102 Rhodobacter sphaeroides ATCC17025 YP_001168932~1 Alphaproteobacteria uid58451
342108 Magnetospirillum magneticum AMB~1 YP_422459.1 Alphaproteobacteria uid58527
391038 Burkholderia phymatum STM815 YP_001861530~1 Betaproteobacteria uid58699
402881 Parvibaculum lavamentivorans DS~1 YP_001414415.1 Alphaproteobacteria uid58739
0.97
311402 Agrobacterium vitis S4 YP_002547364~1 Alphaproteobacteria uid58249
648757 Rhodomicrobium vannielii ATCC17100 YP_004013742.1 Alphaproteobacteria uid43247
391038 Burkholderia phymatum STM815 YP_001856375~1 Betaproteobacteria uid58699
402881 Parvibaculum lavamentivorans DS~1 YP_001412704.1 Alphaproteobacteria uid58739
1082931 Pelagibacterium halotolerans B2 YP_004899313~1 Alphaproteobacteria uid74393
1110502 Tistrella mobilis KA081020~065 YP_006371352.1 Alphaproteobacteria uid167486
644801 Pseudomonas stutzeri RCH2 YP_007242239~1 Gammaproteobacteria uid184342
1430440 Magnetospirillum gryphiswaldense MSR~1v2 YP_008936900.1 Alphaproteobacteria uid232249
246197 Myxococcus xanthus DK1622 YP_632059~1 delta epsilon subdivisions uid58003
862719 Azospirillum lipoferum 4B YP_005038116.1 uid82343
483219 Myxococcus fulvus HW~1 YP_004668486~1 delta epsilon subdivisions uid68443
0.96
1150469 Rhodospirillum photometricum DSM122 YP_005416415.1 Alphaproteobacteria uid159003
1235461 Sinorhizobium meliloti GR4 YP_007193168~1 Alphaproteobacteria uid184823
1036743 Rhodospirillum rubrum F11 YP_006049550.1 Alphaproteobacteria uid162149
1029756 Hyphomicrobium nitrativorans NL23 YP_008865617~1 Alphaproteobacteria uid230615
269796 Rhodospirillum rubrum ATCC11170 YP_428295.1 Alphaproteobacteria uid57655
0.99
573065 Asticcacaulis excentricus CB48 YP_004087195~1 Alphaproteobacteria uid55641
1430440 Magnetospirillum gryphiswaldense MSR~1v2 YP_008936347.1 Alphaproteobacteria uid232249
395963 Beijerinckia indica subspindicaATCC9039 YP_001834066~1 Alphaproteobacteria uid59057
0.964000
342108 Magnetospirillum magneticum AMB~1 YP_420030.1 Alphaproteobacteria uid58527
69328 Pseudomonas sP_VLB120 YP_008763956~1 Gammaproteobacteria uid226717
137722 Azospirillum sP_B510 YP_003448124.1 uid46085
743720 Pseudomonas fulva 12~X YP_004472647~1 Gammaproteobacteria uid67351
414684 Rhodospirillum centenum SW YP_002298099.1 Alphaproteobacteria uid58805
0.95
379731 Pseudomonas stutzeri A1501 YP_001171657~1 Gammaproteobacteria uid58641
1036743 Rhodospirillum rubrum F11 YP_006046335.1 Alphaproteobacteria uid162149
996285 Pseudomonas stutzeri DSM4166 YP_005937733~1 Gammaproteobacteria uid162113
269796 Rhodospirillum rubrum ATCC11170 YP_425158.1 Alphaproteobacteria uid57655
0.99
1.0
96563 Pseudomonas stutzeri ATCC17588=LMG11199 YP_004713392~1 Gammaproteobacteria uid68749
137722 Azospirillum sP_B510 YP_003453089.1 uid46085
316058 Rhodopseudomonas palustris HaA2 YP_484334~1 Alphaproteobacteria uid58439
862719 Azospirillum lipoferum 4B YP_005041325.1 uid82343
0.98
1331671 Pseudomonas putida H8234 YP_008094480~1 Gammaproteobacteria uid208673
395963 Beijerinckia indica subspindicaATCC9039 YP_001832997.1 Alphaproteobacteria uid59057
1211579 Pseudomonas putida NBRC14164 YP_008113880~1 Gammaproteobacteria uid208670
402881 Parvibaculum lavamentivorans DS~1 YP_001413207.1 Alphaproteobacteria uid58739
1215088 Pseudomonas putida HB3267 YP_007229718~1 Gammaproteobacteria uid184078
1110502 Tistrella mobilis KA081020~065 YP_006373894.1 Alphaproteobacteria uid167486
1435044 Pseudomonas monteilii SB3078 YP_008955082~1 Gammaproteobacteria uid232252
0.93
481009 Rickettsia philipii str364D YP_005300397.1 Alphaproteobacteria uid89383
1042876 Pseudomonas putida S16 YP_004702338~1 Gammaproteobacteria uid68747
1105110 Rickettsia australis strCutlack YP_005415369.1 Alphaproteobacteria uid158039
1435058 Pseudomonas monteilii SB3101 YP_008960450~1 Gammaproteobacteria uid232253
1105111 Candidatus Rickettsia amblyommiistrGAT~30V YP_005365058.1 Alphaproteobacteria uid156845
351746 Pseudomonas putida F1 YP_001267656~1 Gammaproteobacteria uid58355
562019 Rickettsia peacockii strRustic YP_002916261.1 Alphaproteobacteria uid59301
1.0
231023 Pseudomonas putida ND6 YP_006389986~1 Gammaproteobacteria uid167583
1032845 Rickettsia heilongjiangensis 054 YP_004764030.1 Alphaproteobacteria uid70839
931281 Pseudomonas putida BIRD~1 YP_005930196~1 Gammaproteobacteria uid162055
941638 Rickettsia slovaca 13~B YP_005065525.1 Alphaproteobacteria uid82369
1196325 Pseudomonas putida DOT~T1E YP_006530776~1 Gammaproteobacteria uid171260
1105109 Rickettsia slovaca strD~CWPP_YP_005426054.1 Alphaproteobacteria uid158159
1064539 Azospirillum brasilense Sp245 YP_005033672~1 uid162161
347255 Rickettsia africae ESF~5 YP_002844978.1 Alphaproteobacteria uid58799
1037409 Bradyrhizobium japonicum USDA6 YP_005607572~1 Alphaproteobacteria uid158851
315456 Rickettsia felis URRWXCal2 YP_247006.1 Alphaproteobacteria uid58331
224911 Bradyrhizobium diazoefficiens USDA110 NP_773356~1 Alphaproteobacteria uid57599
0.99
1105102 Rickettsia rickettsii strColombia YP_005285876.1 Alphaproteobacteria uid86653
224911 Bradyrhizobium diazoefficiens USDA110 NP_772546~1 Alphaproteobacteria uid57599
652620 Rickettsia japonica YH YP_004884601.1 Alphaproteobacteria uid73963
316056 Rhodopseudomonas palustris BisB18 YP_532993~1 Alphaproteobacteria uid58443
1105107 Rickettsia canadensis strCA410 YP_005299308.1 Alphaproteobacteria uid88063
0.93
316058 Rhodopseudomonas palustris HaA2 YP_485765~1 Alphaproteobacteria uid58439
0.98
293613 Rickettsia canadensis strMcKiel YP_001491995.1 Alphaproteobacteria uid58159
754476 Methylophaga nitratireducenticrescens YP_006296464~1 Gammaproteobacteria uid162947
272947 Rickettsia prowazekii strMadridE NP_220615.1 Alphaproteobacteria uid61565
1148 Synechocystis sP_PCC6803 YP_007459877~1 Cyanobacteria uid189748
1290428 Rickettsia prowazekii strBreinl YP_007750668.1 Alphaproteobacteria uid196851
1148 Synechocystis sP_PCC6803 NP_942444~1 Cyanobacteria uid57659
0.99
1105097 Rickettsia prowazekii strDachau YP_005407014.1 Alphaproteobacteria uid158057
114615 Bradyrhizobium sP_ORS278 YP_001205486~1 Alphaproteobacteria uid58941
1105098 Rickettsia prowazekii strGvV257 YP_005404752.1 Alphaproteobacteria uid158051
288000 Bradyrhizobium sP_BTAi1 YP_001240195~1 Alphaproteobacteria uid58505
0.92
0.99
1105099 Rickettsia prowazekii strRpGvF24 YP_005413672.1 Alphaproteobacteria uid158065
288000 Bradyrhizobium sP_BTAi1 YP_001241215~1 Alphaproteobacteria uid58505
1290427 Rickettsia prowazekii strNMRCMadridE YP_007750201.1 Alphaproteobacteria uid196850
316056 Rhodopseudomonas palustris BisB18 YP_530260~1 Alphaproteobacteria uid58443
449216 Rickettsia prowazekii strRp22 YP_005998639.1 Alphaproteobacteria uid161945
114615 Bradyrhizobium sP_ORS278 YP_001207640~1 Alphaproteobacteria uid58941
0.91
1105095 Rickettsia prowazekii strKatsinyian YP_005406173.1 Alphaproteobacteria uid158055
288000 Bradyrhizobium sP_BTAi1 YP_001242091~1 Alphaproteobacteria uid58505
1105094 Rickettsia prowazekii strChernikova YP_005405327.1 Alphaproteobacteria uid158053
288000 Bradyrhizobium sP_BTAi1 YP_001243575~1 Alphaproteobacteria uid58505
1105096 Rickettsia prowazekii strBuV67~CWPP_YP_005412834.1 Alphaproteobacteria uid158063
1245469 Bradyrhizobium oligotrophicum S58 YP_007516805~1 Alphaproteobacteria uid192186
414684 Rhodospirillum centenum SW YP_002298909.1 Alphaproteobacteria uid58805
0.94
114615 Bradyrhizobium sP_ORS278 YP_001208913~1 Alphaproteobacteria uid58941
342108 Magnetospirillum magneticum AMB~1 YP_419930.1 Alphaproteobacteria uid58527
450851 Phenylobacterium zucineum HLK1 YP_002130918~1 Alphaproteobacteria uid58959
1064539 Azospirillum brasilense Sp245 YP_005029913.1 uid162161
0.90
366602 Caulobacter sP_K31 YP_001685174~1 Alphaproteobacteria uid58551
0.93
1117321 Azospirillum brasilense CBG497 AZCBGv5 40096ID10457916 uid000000
272630 Methylobacterium extorquens AM1 YP_002962727~1 Alphaproteobacteria uid57605
0.96
137722 Azospirillum sP_B510 YP_003447561.1 uid46085
1.0
441620 Methylobacterium populi BJ001 YP_001926464~1 Alphaproteobacteria uid58937
862719 Azospirillum lipoferum 4B YP_005040059.1 uid82343
0.94
441620 Methylobacterium populi BJ001 YP_001922861~1 Alphaproteobacteria uid58937
1430440 Magnetospirillum gryphiswaldense MSR~1v2 YP_008937763.1 Alphaproteobacteria uid232249
426117 Methylobacterium sP_4~46 YP_001771481~1 Alphaproteobacteria uid58843
342108 Magnetospirillum magneticum AMB~1 YP_422213.1 Alphaproteobacteria uid58527
0.99
0.95
440085 Methylobacterium extorquens CM4 YP_002418917~1 Alphaproteobacteria uid58933
1110502 Tistrella mobilis KA081020~065 YP_006371346.1 Alphaproteobacteria uid167486
441620 Methylobacterium populi BJ001 YP_001922769~1 Alphaproteobacteria uid58937
1430440 Magnetospirillum gryphiswaldense MSR~1v2 YP_008936693.1 Alphaproteobacteria uid232249
272630 Methylobacterium extorquens AM1 YP_002961267~1 Alphaproteobacteria uid57605
137722 Azospirillum sP_B510 YP_003452479.1 uid46085
419610 Methylobacterium extorquens PA1 YP_001637518~1 Alphaproteobacteria uid58821
862719 Azospirillum lipoferum 4B YP_004975186.1 uid82343
0.99
661410 Methylobacterium extorquens DM4 YP_003065765~1 Alphaproteobacteria uid61617
137722 Azospirillum sP_B510 YP_003450084.1 uid46085
0.99
137722 Azospirillum sP_B510 YP_003452479~1 uid46085
862719 Azospirillum lipoferum 4B YP_004973569.1 uid82343
0.96
862719 Azospirillum lipoferum 4B YP_004975186~1 uid82343
0.98
AZOLI p10058
137722 Azospirillum sP_B510 YP_003450084~1 uid46085
402881 Parvibaculum lavamentivorans DS~1 YP_001412976.1 Alphaproteobacteria uid58739
862719 Azospirillum lipoferum 4B YP_004973569~1 uid82343
1150469 Rhodospirillum photometricum DSM122 YP_005417915.1 Alphaproteobacteria uid159003
0.96
AZOLI p10058
0.96
648757 Rhodomicrobium vannielii ATCC17100 YP_004011328.1 Alphaproteobacteria uid43247
450851 Phenylobacterium zucineum HLK1 YP_002131444~1 Alphaproteobacteria uid58959
573065 Asticcacaulis excentricus CB48 YP_004087774.1 Alphaproteobacteria uid55641
460265 Methylobacterium nodulans ORS2060 YP_002502721~1 Alphaproteobacteria uid59023
137722 Azospirillum sP_B510 YP_003449797.1 uid46085
460265 Methylobacterium nodulans ORS2060 YP_002498720~1 Alphaproteobacteria uid59023
0.99
862719 Azospirillum lipoferum 4B YP_005037760.1 uid82343
0.99
450851 Phenylobacterium zucineum HLK1 YP_002128623~1 Alphaproteobacteria uid58959
1064539 Azospirillum brasilense Sp245 YP_005032808.1 uid162161
0.90
335659 Bradyrhizobium sP_S23321 YP_005453763~1 Alphaproteobacteria uid158167
1117321 Azospirillum brasilense CBG497 AZCBGv5 10002ID10457617 uid000000
748247 Azoarcus sP_KH32C YP_007550025~1 Betaproteobacteria uid193704
137722 Azospirillum sP_B510 YP_003448078.1 uid46085
0.99
1037409 Bradyrhizobium japonicum USDA6 YP_005607301~1 Alphaproteobacteria uid158851
862719 Azospirillum lipoferum 4B YP_005038317.1 uid82343
573065 Asticcacaulis excentricus CB48 YP_004087039~1 Alphaproteobacteria uid55641
1064539 Azospirillum brasilense Sp245 YP_005032440.1 uid162161
1037911 Pseudomonas fluorescens A506 YP_006323676~1 Gammaproteobacteria uid165185
1117321 Azospirillum brasilense CBG497 AZCBGv5 490058ID10460263 uid000000
0.99
743720 Pseudomonas fulva 12~X YP_004473266~1 Gammaproteobacteria uid67351
137722 Azospirillum sP_B510 YP_003447877.1 uid46085
0.99
316273 Xanthomonas campestris pvvesicatoriastr85~10 YP_364485~1 Gammaproteobacteria uid58321
862719 Azospirillum lipoferum 4B YP_005038215.1 uid82343
0.99
1137651 Xanthomonas citri subspcitriAw12879 YP_007650222~1 Gammaproteobacteria uid194444
1029756 Hyphomicrobium nitrativorans NL23 YP_008867100.1 Alphaproteobacteria uid230615
1.0
190486 Xanthomonas axonopodis pvcitristr306 NP_642870~1 Gammaproteobacteria uid57889
0.94
1.0
582899 Hyphomicrobium denitrificans ATCC51888 YP_003755986.1 Alphaproteobacteria uid50325
1304892 Xanthomonas axonopodis Xac29~1 YP_007637004~1 Gammaproteobacteria uid193774
717785 Hyphomicrobium sP_MC1 YP_004676108.1 Alphaproteobacteria uid68453
0.98
366649 Xanthomonas fuscans subspfuscans YP_008639284~1 Gammaproteobacteria uid222814
670307 Hyphomicrobium denitrificans 1NES1 YP_007914405.1 Alphaproteobacteria uid179904
0.96
360094 Xanthomonas oryzae pvoryzaePXO99A YP_001914487~1 Gammaproteobacteria uid59131
426355 Methylobacterium radiotolerans JCM2831 YP_001754427.1 Alphaproteobacteria uid58845
981368 Xanthomonas axonopodis pvcitrumeloF1 YP_004852094~1 Gammaproteobacteria uid73179
272630 Methylobacterium extorquens AM1 YP_002961889.1 Alphaproteobacteria uid57605
0.93
342109 Xanthomonas oryzae pvoryzaeMAFF311018 YP_452017~1 Gammaproteobacteria uid58547
0.99
661410 Methylobacterium extorquens DM4 YP_003066692.1 Alphaproteobacteria uid61617
0.93
383407 Xanthomonas oryzae pvoryzicolaBLS256 YP_005628219~1 Gammaproteobacteria uid54411
440085 Methylobacterium extorquens CM4 YP_002419719.1 Alphaproteobacteria uid58933
190485 Xanthomonas campestris pvcampestrisstrATCC33913 NP_637775~1 Gammaproteobacteria uid57887
419610 Methylobacterium extorquens PA1 YP_001638388.1 Alphaproteobacteria uid58821
509169 Xanthomonas campestris pvcampestrisstrB100 YP_001903150~1 Gammaproteobacteria uid61643
441620 Methylobacterium populi BJ001 YP_001923561.1 Alphaproteobacteria uid58937
0.99
990315 Xanthomonas campestris pvraphani756C YP_005637715~1 Gammaproteobacteria uid159539
1245469 Bradyrhizobium oligotrophicum S58 YP_007512529.1 Alphaproteobacteria uid192186
314565 Xanthomonas campestris pvcampestrisstr8004 YP_242777~1 Gammaproteobacteria uid57595
114615 Bradyrhizobium sP_ORS278 YP_001206686.1 Alphaproteobacteria uid58941
1245469 Bradyrhizobium oligotrophicum S58 YP_007510402~1 Alphaproteobacteria uid192186
288000 Bradyrhizobium sP_BTAi1 YP_001239459.1 Alphaproteobacteria uid58505
288000 Bradyrhizobium sP_BTAi1 YP_001237047~1 Alphaproteobacteria uid58505
224911 Bradyrhizobium diazoefficiens USDA110 NP_770378.1 Alphaproteobacteria uid57599
0.99
114615 Bradyrhizobium sP_ORS278 YP_001208470~1 Alphaproteobacteria uid58941
335659 Bradyrhizobium sP_S23321 YP_005451817.1 Alphaproteobacteria uid158167
757424 Herbaspirillum seropedicae SmR1 YP_003775860~1 Betaproteobacteria uid50427
0.99
1037409 Bradyrhizobium japonicum USDA6 YP_005610992.1 Alphaproteobacteria uid158851
391038 Burkholderia phymatum STM815 YP_001858578~1 Betaproteobacteria uid58699
316057 Rhodopseudomonas palustris BisB5 YP_569182.1 Alphaproteobacteria uid58441
419610 Methylobacterium extorquens PA1 YP_001639119~1 Alphaproteobacteria uid58821
316058 Rhodopseudomonas palustris HaA2 YP_487002.1 Alphaproteobacteria uid58439
661410 Methylobacterium extorquens DM4 YP_003067865~1 Alphaproteobacteria uid61617
258594 Rhodopseudomonas palustris CGA009 NP_947317.1 Alphaproteobacteria uid62901
0.99
272630 Methylobacterium extorquens AM1 YP_002962682~1 Alphaproteobacteria uid57605
652103 Rhodopseudomonas palustris DX~1 YP_004109836.1 Alphaproteobacteria uid43327
440085 Methylobacterium extorquens CM4 YP_002420722~1 Alphaproteobacteria uid58933
395960 Rhodopseudomonas palustris TIE~1 YP_001991181.1 Alphaproteobacteria uid58995
426117 Methylobacterium sP_4~46 YP_001771637~1 Alphaproteobacteria uid58843
1031710 Oligotropha carboxidovorans OM4 YP_005950540.1 Alphaproteobacteria uid162135
0.94
426355 Methylobacterium radiotolerans JCM2831 YP_001756226~1 Alphaproteobacteria uid58845
0.93
504832 Oligotropha carboxidovorans OM5 YP_002289519.1 Alphaproteobacteria uid59155
272630 Methylobacterium extorquens AM1 YP_002964366~1 Alphaproteobacteria uid57605
504832 Oligotropha carboxidovorans OM5 YP_004632476.1 Alphaproteobacteria uid72795
419610 Methylobacterium extorquens PA1 YP_001640598~1 Alphaproteobacteria uid58821
323098 Nitrobacter winogradskyi Nb~255 YP_317826.1 Alphaproteobacteria uid58295
0.1
0.1
1.0
441620 Methylobacterium populi BJ001 YP_001926022~1 Alphaproteobacteria uid58937
0.95
323097 Nitrobacter hamburgensis X14 YP_576751.1 Alphaproteobacteria uid58293
661410 Methylobacterium extorquens DM4 YP_003069406~1 Alphaproteobacteria uid61617
316056 Rhodopseudomonas palustris BisB18 YP_531937.1 Alphaproteobacteria uid58443
440085 Methylobacterium extorquens CM4 YP_002422209~1 Alphaproteobacteria uid58933
316055 Rhodopseudomonas palustris BisA53 YP_780902.1 Alphaproteobacteria uid58445
